# Transcriptome comparative analysis of ovarian follicles reveals the key genes and signaling pathways implicated in hen egg production

**DOI:** 10.1186/s12864-021-08213-w

**Published:** 2021-12-15

**Authors:** Xue Sun, Xiaoxia Chen, Jinghua Zhao, Chang Ma, Chunchi Yan, Simushi Liswaniso, Rifu Xu, Ning Qin

**Affiliations:** 1 Joint Laboratory of Modern Agricultural Technology International Cooperation, Ministry of Education, Jilin Agricultural University, Changchun, 130118 China; 2 Department of Animal Genetics, Breeding and Reproduction, College of Animal Science and Technology, Jilin Agricultural University, Changchun, 130118 China

**Keywords:** Ovarian follicle, Transcriptome, *NDUFAB1*, *GABRA1*, Egg production

## Abstract

**Background:**

Ovarian follicle development plays an important role in determination of poultry egg production. The follicles at the various developmental stages possess their own distinct molecular genetic characteristics and have different biological roles in chicken ovary development and function. In the each stage, several genes of follicle-specific expression and biological pathways are involved in the vary-sized follicular development and physiological events. Identification of the pivotal genes and signaling pathways that control the follicular development is helpful for understanding their exact regulatory functions and molecular mechanisms underlying egg-laying traits of laying hens.

**Results:**

The comparative mRNA transcriptomic analysis of ovarian follicles at three key developmental stages including slow growing white follicles (GWF), small yellow follicles (SYF) of recruitment into the hierarchy, and differentiated large yellow follicles (LYF), was accomplished in the layers with lower and higher egg production. Totally, 137, 447, and 229 of up-regulated differentially expressed genes (DEGs), and 99, 97, and 157 of down-regulated DEGs in the GWF, SYF and LYF follicles, including *VIPR1*, *VIPR2*, *ADRB2*, and *HSD17B1* were identified, respectively. Moreover, *NDUFAB1* and *GABRA1* genes, two most promising candidates potentially associated with egg-laying performance were screened out from the 13 co-expressed DEGs in the GWF, SYF and LYF samples. We further investigated the biological effects of *NDUFAB1* and *GABRA1* on ovarian follicular development and found that *NDUFAB1* promotes follicle development by stimulating granulosa cell (GC) proliferation and decreasing cell apoptosis, increases the expression of CCND1 and BCL-2 but attenuates the expression of caspase-3, and facilitates steroidogenesis by enhancing the expression of STAR and CYP11A1. In contrast, *GABRA1* inhibits GC proliferation and stimulates cell apoptosis, decreases the expression of CCND1, BCL-2, STAR, and CYP11A1 but elevates the expression of caspase-3. Furthermore, the three crucial signaling pathways such as PPAR signaling pathway, cAMP signaling pathway and neuroactive ligand-receptor interaction were significantly enriched, which may play essential roles in ovarian follicle growth, differentiation, follicle selection, and maturation.

**Conclusions:**

The current study provided new molecular data for insight into the regulatory mechanism underlying ovarian follicle development associated with egg production in chicken.

**Supplementary Information:**

The online version contains supplementary material available at 10.1186/s12864-021-08213-w.

## Background

Egg production initiates from the follicle prehierarchical and hierarchical development, maturation, and finally ovulates from the hen ovary, which is governed by the hypothalamic–pituitary–ovarian axis [1–3]. Ovarian follicle development plays a key role in the egg production capacity, which is characterized by a well-organized follicular hierarchy in high production egg-laying layers. In chickens with low egg-laying rates, e.g., the broiler breeder hen, follicular development is not well-organized, which leads to reduced productivity [1]. Generally, ovarian follicles can be categorized by size or/and according to color (white or yellow), into at least four types, including small white resting follicles (less than 2 mm diameter), slow growing white follicles (GWF, from 2 mm up to 6 mm diameter), small yellow follicles (SYF, 6 up to 8 mm) of recruitment into the follicular hierarchy (at the stage of follicle selection), and large yellow follicles (LWF) at the differentiated preovulatory stage, being 9 to 12 mm in diameter and five to six hierarchical follicles of increased sizes (from F6 to F1) in hen ovary [4, 5]. In the different phases of follicular development, many divergent biological processes affect oocyte growth, and proliferation and differentiation of granulosa and theca cells within the various-sized follicles [4, 6, 7]. Moreover, a plethora of ovarian paracrine and autocrine factors was involved in regulation of the follicle development and its function as well as the positive or negative controls through the endocrinal hormones from the hypothalamus and pituitary, including gonadotropin releasing hormone (GNRH), gonadotropin inhibitory hormone (GNIH), and follicle stimulating hormone (FSH) [1, 2, 8]. Within the ovary, the most representative hormones and growth factors such as steroidogenic-related enzymes steroidogenic acute regulatory protein (STAR), hydroxysteroid (17beta) dehydrogenase 1 (HSD17B1) and cytochrome P450 side-chain cleavage (P450scc/CYP11A1), intra-ovarian hormones progesterone (P4), estradiol (E2) and anti-müllerian hormone (AMH), cell proliferation or apoptosis-related factors Bcl-2, cyclin D1 (CCND1) and caspase-3 (CASP3), which have indispensable effects on follicular development, follicle selection or atresia, finally on their preovulatory development and ovulation, making the early developmental differences of ovarian follicles and egg production capacity in adult layers highly correlated, have been intensively investigated [9–13]. Undoubtedly, follicular size has a close relationship with its developmental biology, physiological function, and molecular regulation. It has been drawing so much attention to extensive exploration of the key genes and signaling pathways to be implicated in ovarian follicle development and potentially associated with egg production in chicken.

To intensively explore the molecular regulatory mechanisms underlying follicle development for better understanding egg production capacity, previous studies on transcriptome analyses of ovarian follicles, granulosa cells and theca cells of follicles as well as hypothalamus, pituitary gland and ovary, have revealed many genes involved in ovarian follicular development and follicle selection, which potentially correlate with the high capacity for egg production in chicken, goose, duck, and turkey. It has been reported that 20 differentially expressed transcripts (e.g., *FTH1*, *TB*, *EEF1A1*, *TXN*, *ANXA2*, *ING4*, and *ACADL*) associated with high rates of egg production in chicken ovarian follicles were screened out by cDNA microarray data analysis [14]. Several differentially expressed transcripts implicated in steroidogenesis (*STAR*, *HSD3B*, *CYP11A1*, and *CYP19*), paracrine signaling (*PCSK6*, *KITL* and *WNT4*) and transcription (*FOXO3*, *FOXL2* and *WT1*) were identified by transcriptome analysis of the small ovarian follicles 0.5, 1, and 2 mm in diameter after oocyte removal in chicken, which may be important during early avian follicular development [15]. Transcriptomic analysis of single small yellow follicles demonstrated that *Wnt4* takes part in the regulation of chicken follicle selection [16]. Transcriptomic analysis found 855 candidates differentially expressed between small yellow follicles (SYF, 6–8 mm in diameter) and F6 follicles in laying hens, including *VLDLR1*, *WIF1*, *NGFR*, *AMH*, *BMP15*, *GDF6* and *MMP13*. They might play certain roles in chicken follicle selection [17]. Integrated transcriptomic analysis on chicken SYF differing in follicle stimulating hormone (FSH) receptor expression discovered 467 differentially expressed genes (DEGs). And sosondowah ankyrin repeat domain family member A (*SOWAHA*) gene was confirmed to affect the expression of genes involved in chicken follicle selection and to inhibit the proliferation of granulosa cells [18]. Moreover, RNA sequencing was used to analyze mRNAs and long noncoding RNAs (lncRNAs) from granulosa cells of SYFs from Jinghai Yellow chickens in exposure to red light and white light groups. One thousand one hundred eighty-two DEGs were identified and the integrated network analysis shown that several of them were involved in follicular development through steroid hormone synthesis, oocyte meiosis, and the PI3K-Akt signaling pathway [19].

Additionally, similar studies on transcriptome analysis of ovarian follicles were also reported in goose, duck and turkey. It was published that transcriptomic profiling identifies 688 DEGs in Huoyan goose ovaries (including the small and large yellow follicles) between the laying period and ceased period, in which the 12 validated genes, i.e., *PGR*, *INSR*, *NPY1R*, *ESRRB*, *MEL1C*, *VIPR2*, *LHCGR*, *SCG2*, *GHR*, *STAR*, *HSD3B2*, and *CYP11A1*, are mainly involved in the signal transduction pathways for reproduction regulation, such as steroid hormone biosynthesis, GnRH signaling pathways, oocyte meiosis, progesterone-mediated oocyte maturation, steroid biosynthesis, calcium signaling pathways, and G-protein coupled receptor signaling pathway [20]. Ren showed seven key DEGs, including *EPOX*, *StAR*, *CYP17*, *3β-HSD*, *CYP1B1*, *CYP19A1*, and *SR-B1*, were related to Peking duck ovarian follicle development [21]. Moreover, transcriptome analysis of the granulosa layer of the largest follicle (F1) and fifth largest follicle (F5), theca interna layer of F5 follicle, and small white follicle samples from low and high egg producing turkey hens revealed 12,221 DEGs were identified, and among them, several of the most promising candidates and signaling pathways may be mainly involved in differential regulation of the hypothalamo-pituitary-thyroid axis, particularly thyroid hormone transporters and thyroid hormone receptors, and of estradiol signaling [22]. However, the pivotal genes and signaling pathways controlling the development of various-sized ovarian follicles in chicken, and their exact physiological roles and regulatory mechanisms in egg production remain largely unclear yet.

The Jilin Black (JB) chicken, an indigenous Chinese breed (dual-purpose type for egg and meat production), is characterized by high quality meat, but a low rate of egg production, low laying peak, and strong broodiness [23]. In contrast, the Lohmann Brown (LB) layer, a commercial egg-laying breed, is characterized as possessing a high rate of egg production, high laying peak, and great consistency in laying performance [24]. The differences in the egg production capacity between the JB and LB breeds may, therefore, serve as a proper basis for discovering potential key genes involved in egg production and for further investigating the molecular mechanism underlying formation of egg-laying traits in chicken.

To find the most potential genes and signaling pathways associated with egg production, comparative transcriptome analysis of ovarian GWF, SYF and LYF follicles between the JB and LB breeds was implemented. The present study aims to identify key functional genes implicated in ovarian follicular growth and development (including follicle selection) that directly contribute to egg-laying production. These results laid a molecular foundation for further elucidating the regulatory mechanisms of the promising genes in ovary development, and the pivotal signaling pathways in formation of chicken egg-laying traits.

## Results

### Comparison of egg-laying production related traits between JB and LB hens

Performance of egg-laying production related traits in JB and LB hens was evaluated, including age at first egg (AF), average body weight at 21 and 66 weeks of age (21BW and 66 BW), egg weight at 66 weeks of age (EW), hen-housed egg production (HH) at 72 weeks of age, and egg production (total egg numbers per hen) rate at 21, 30, 43, 57 and 66 weeks of age (21EN, 30EN, 43EN, 57EN and 66EN). As shown in Table 1, the AF, which is the days of age when egg production rate per day reaches 50%, was significantly earlier in LB than JB hens (*P* < 0.001). HH at 72 weeks of age was higher in LB than JB hens (*P* < 0.001). Moreover, egg production rates at 21, 30, 43, 57 and 66 weeks of age were also higher in LB when compared to JB hens (*P* < 0.001).

**Table 1:** Comparison of egg productions between JB and LB hens

Breed	AF	21BW	66BW	EW	HH	21EN	30EN	43EN	57EN	66EN
LB(*n* = 720)	141.2^*^	1.71 ± 0.13	1.98 ± 0.45	64.7 ± 2.26	289.6^*^	53.49^*^	93.82^*^	88.56^*^	83.75^*^	74.34^*^
JB(*n* = 458)	206.5	1.78 ± 0.19	2.21 ± 0.76	62.9 ± 4.48	152.7	11.32	54.62	64.39	57.18	46.27

*AF* age at first egg (days), *BW* average body weight (kg) at 21 and 66 weeks of age, *EW* average egg weight (g) at 66 weeks of age, *HH* average hen-housed egg production (egg numbers) at 72 weeks of age, *EN* average egg production rate (%) at 21, 30, 43, 57, and 66 weeks of age. The superscript asterisks

* indicates significant difference of the means at the level, *P* < 0.001

### Characteristics of RNA sequencing data

**Fig. 1:**
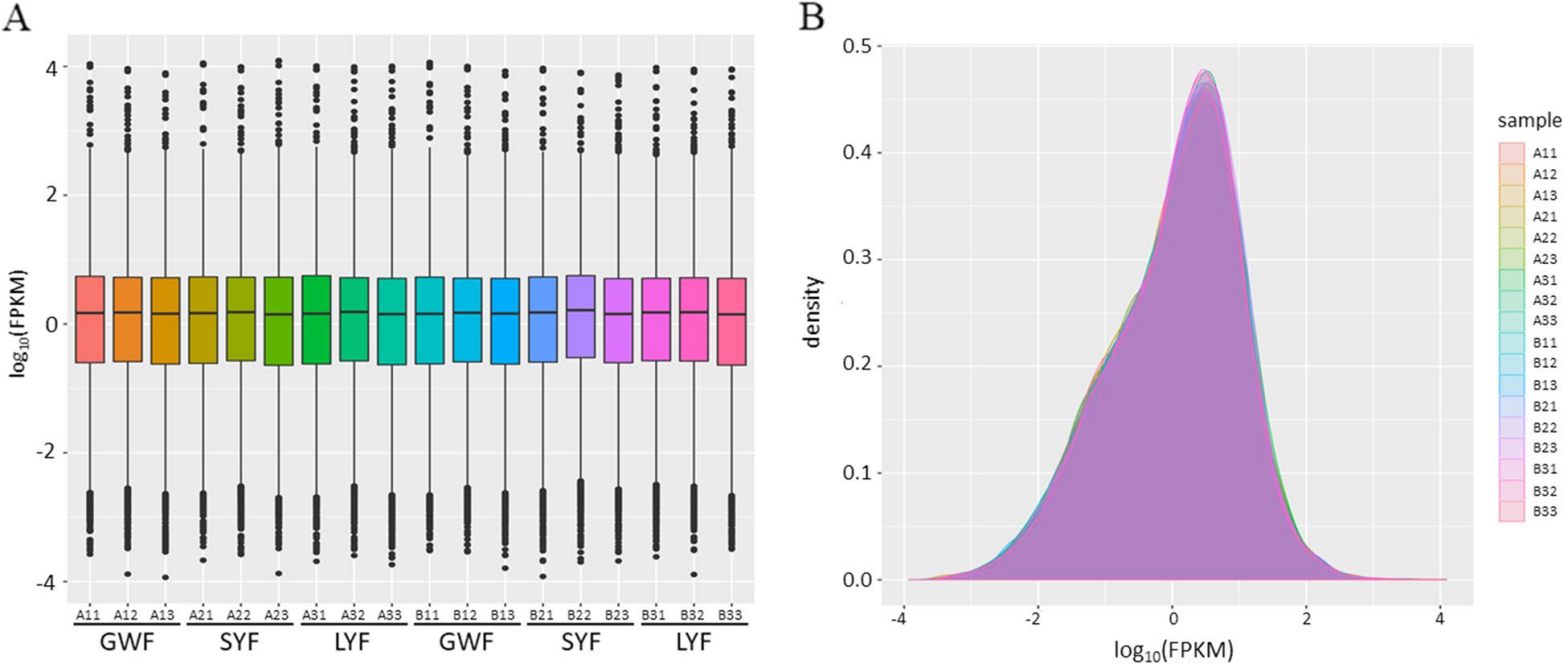
FPKM distribution of transcript expression levels in the various-sized ovarian follicle samples. **A** Box plot of FPKM distribution with logarithmic values of FPKM on the vertical axis and different follicle samples on the horizontal axis. **B** Density plot of expression distribution with density values on the vertical axis and logarithmic values of FPKM on the horizontal axis. A1, A2, and A3, indicate GWF, SYF, and LYF follicle samples of LB hens, respectively; B1, B2, and B3, indicate GWF, SYF, and LYF samples of JB hens, respectively

Nine cDNA libraries of the GWF, SYF and LYF samples from JB and LB layers were constructed, respectively. In total, 822,883,832 sequence reads were obtained, and with an average of 45,430,781 clean reads each sample (Supplementary Table S1). On average, the clean data of high quality accounted for 99.09% of the sequences of raw reads, 85.59% of the clean reads were mapped to the reference genome of *gallus gallus* and 81.97% reads were mapped uniquely. The dynamic range of the expression values was estimated and exhibited as a box plot of logarithmic transformed FPKM values for each sample separately (Fig. 1A), and the FPKM density distribution is presented in Fig. 1B.

### Identification of differentially expressed genes and signaling pathway enrichment

**Fig. 2:**
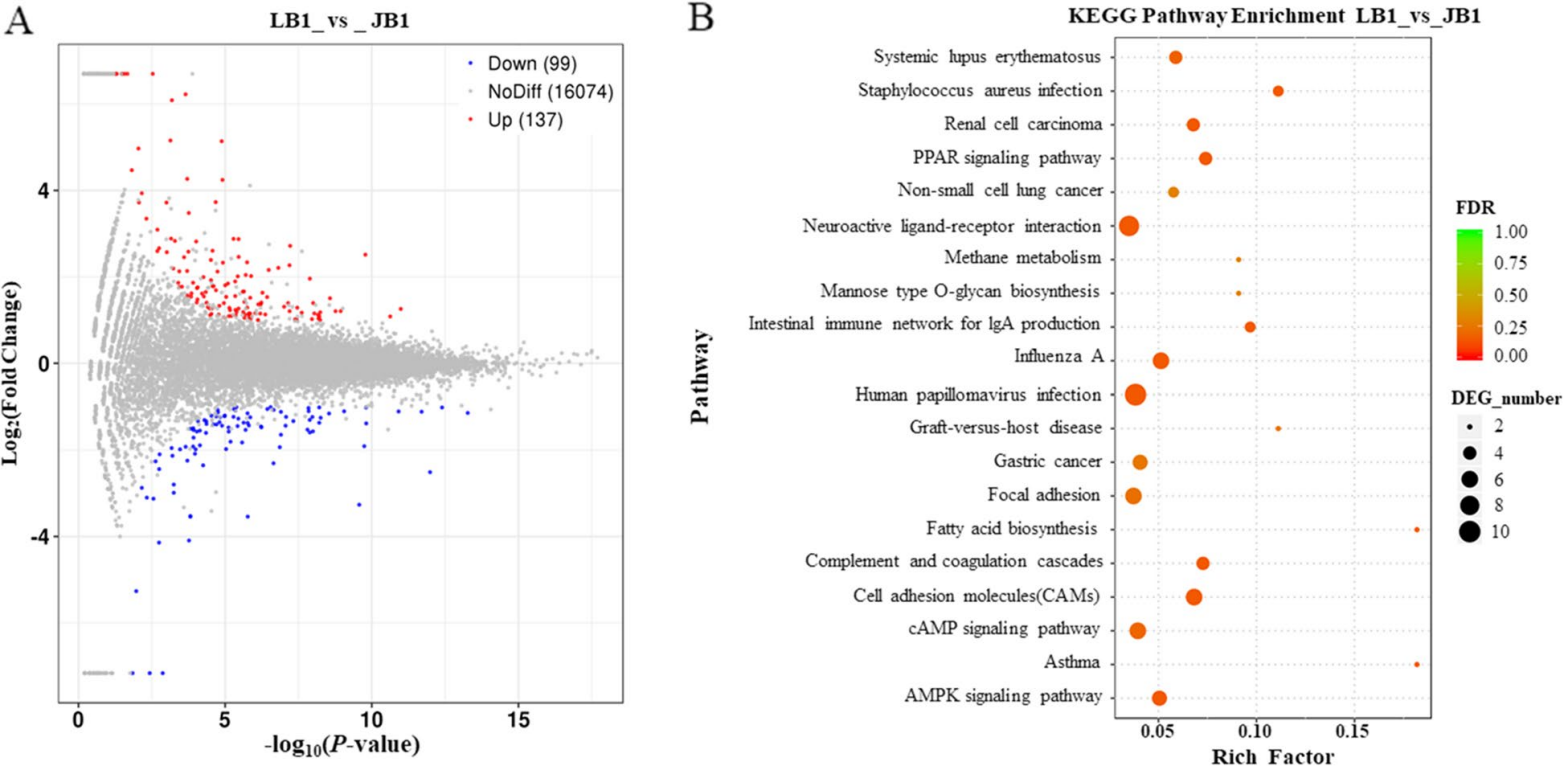
Scatterplot of annotated differently expressed genes and enriched signaling pathways in GWF follicles between JB and LB chickens. **A** MA plot of differently expressed genes in GWF follicles between JB and LB samples. Y-axis: The logarithmic value (log_2_FoldChange) of the multiple of the difference in the expression level of a gene between two samples; X-axis: The negative pair value of the error detection rate (−log_10_FDR). Red dots represent significantly up-regulated genes, blue dots indicate down-regulated genes, and grey dots imply non-differentially expressed genes (*P*-value adjusted for multiple testing < 0.05). JB1, GWF follicle samples of JB hens; LB1, GWF samples of LB hens. **B** Bubble chart of top 20 signaling pathway enrichment with KEGG pathway on the vertical axis and rich impact values on the horizontal axis. Color indicates pathway with the *P*-values. Bubble size represents the ratio of DEG number to the total number of genes enriched on the pathway

In this study, differentially expressed genes (DEGs) in the follicles of GWF, SYF and LYF between JB and LB hens were identified (The relevant datasets have been published by our group and are available at PRJNA669967 and PRJNA670553 in NCBI), respectively. In GWF follicles, 137 up-regulated and 99 down-regulated DEGs were revealed by comparison of JB with LB chickens (Fig. 2A; |log_2_FoldChange| > 1, *P* < 0.05). Among them, the most important potential genes causing development of the undifferentiated follicles, which may be associated with egg production performance, were uncovered such as *GABRA1*, *ADGRD1*, *VIPR2*, *APOD*, *BRINP1*, *PAK5*, *GPR65*, *RYR2*, *ZPBP*, *PIK3R1*, *EDIL3*, *TP53I3*, *BLK*, *POSTN*, *PERP1*, *HACD4*, *ZP1*, *WNT2B*, *CYP4F22*, *ADRB2*, *STARD4*, *CREB3L3*, *ACSBG2*, *GPER1*, *NDUFAB1*, *NCAM2*, *WISP1*, *MC2R*, *LOC424014*, and *RXRG* (Table 2). Moreover, the significant top 20 signaling pathways were displayed (Fig. 2B), including PPAR signaling pathway, neuroactive ligand-receptor interaction, cAMP signaling pathway, fatty acid biosynthesis, cell adhesion molecules (CAMs), and AMPK signaling pathway.

**Table 2:** Characterization of partial DEGs in GWF follicles of JB and LB chickens

Name	Description	Regulation	Chr.	Log_2_FC	*P*-value
*GABRA1*	gamma-aminobutyric acid type A receptor alpha1 subunit	up	13	2.2084	4.8499E-07
*ADGRD1*	adhesion G protein-coupled receptor D1	up	15	1.6618	0.0364
*VIPR2*	vasoactive intestinal peptide receptor 2	up	2	2.2675	1.7403E-07
*APOD*	apolipoprotein D	up	9	2.1226	3.8281E-05
*BRINP1*	BMP/retinoic acid inducible neural specific 1	up	17	2.1930	0.0195
*PAK5*	p21 (RAC1) activated kinase 5	up	3	1.0703	0.0047
*GPR65*	G protein-coupled receptor 65	up	5	1.5475	0.0103
*RYR2*	ryanodine receptor 2	up	3	2.3283	0.0048
*ZPBP*	zona pellucida binding protein	up	2	3.0925	0.0042
*PIK3R1*	phosphoinositide-3-kinase regulatory subunit 1	up	Z	1.9585	0.0419
*EDIL3*	EGF like repeats and discoidin domains 3	up	Z	2.0187	0.0052
*TP53I3*	tumor protein p53 inducible protein 3	up	3	2.3365	0.0311
*BLK*	BLK proto-oncogene, Src family tyrosine kinase	up	3	1.2293	0.0085
*POSTN*	periostin	up	1	1.0831	0.0325
*PERP1*	PERP1, TP53 apoptosis effector	up	3	5.1568	5.0997E-05
*HACD4*	3-hydroxyacyl-CoA dehydratase 4	up	Z	2.8745	0.0171
*ZP1*	zona pellucida glycoprotein 1	up	5	1.2576	0.0337
*WNT2B*	Wnt family member 2B	up	26	2.5831	0.0003
*CYP4F22*	Cytochrome P450 family 4 subfamily member 22	up	30	1.5370	0.0018
*ADRB2*	adrenoceptor beta 2	up	13	1.5427	0.0292
*STARD4*	StAR related lipid transfer domain containing 4	down	Z	−1.3823	3.0970E-09
*CREB3L3*	cAMP responsive element binding protein 3 like 3	down	28	−1.8905	0.0031
*ACSBG2*	acyl-CoA synthetase bubblegum family member 2	down	28	−1.0163	1.6471E-05
*GPER1*	G protein-coupled estrogen receptor 1	down	14	−1.9229	0.0171
*NDUFAB1*	NADH: ubiquinone oxidoreductase subunit AB1	down	14	−1.2524	0.0062
*NCAM2*	neural cell adhesion molecule 2	down	1	−1.9101	0.0049
*WISP1*	WNT1 inducible signaling pathway protein 1	down	2	−1.2337	4.4686E-06
*MC2R*	melanocortin 2 receptor	down	2	−2.7876	4.9574E-05
*LOC424014*	putative methyltransferase DDB_G0268948	down	7	−3.5398	5.0597E-20
*RXRG*	retinoid X receptor gamma	down	8	−1.4435	0.0017

**Fig. 3:**
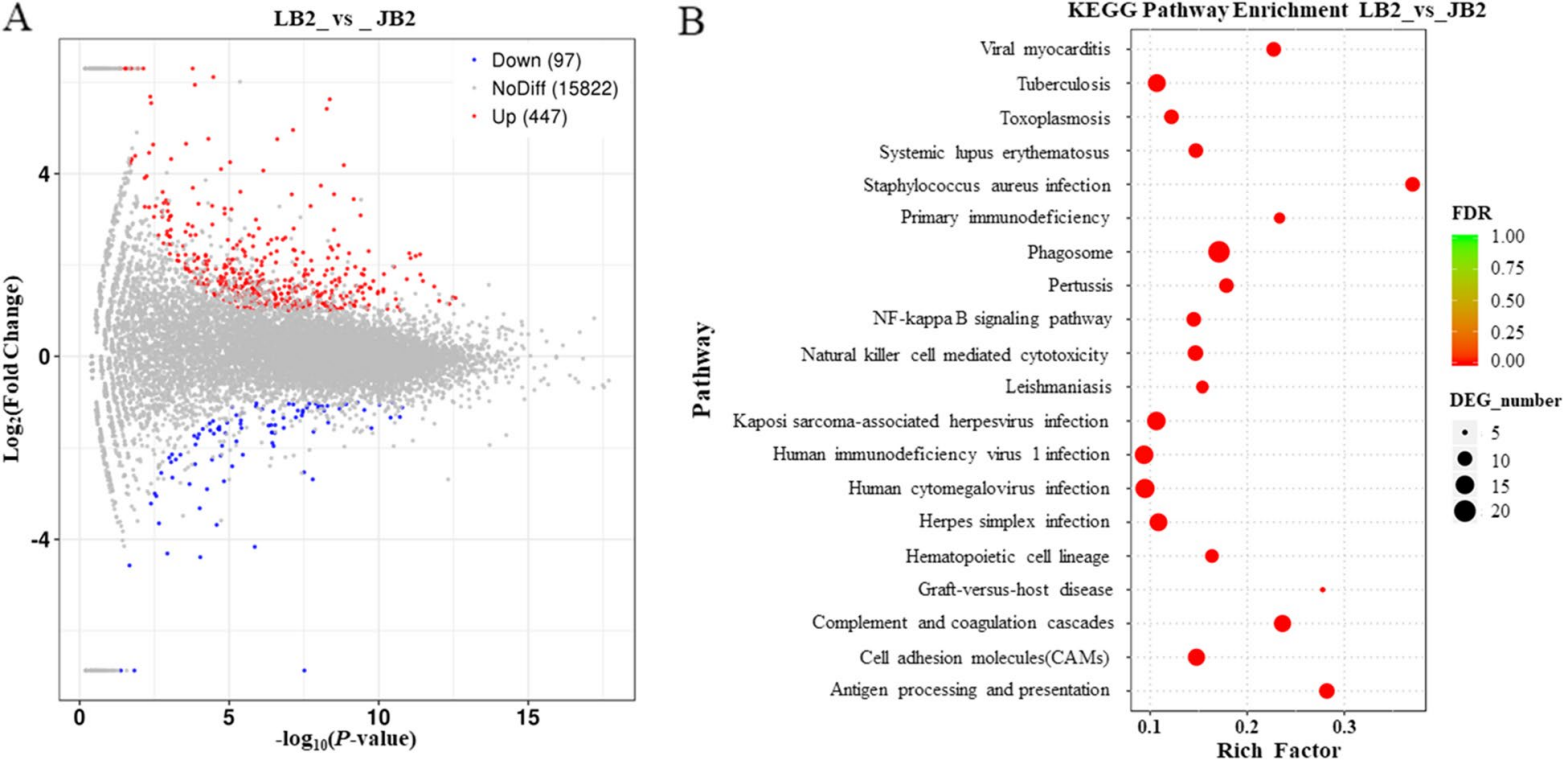
Scatter plot of annotated differently expressed genes and enriched signaling pathways in SYF follicles between JB and LB chickens. **A** MA plot of differently expressed genes in SYF follicles between JB and LB samples. JB2, SYF follicle samples of JB hens; LB2, SYF samples of LB hens. **B** Bubble chart of top 20 of KEGG pathway enrichment

In SYF follicles, 447 of up-regulated and 97 of down-regulated DEGs were discovered in comparison of JB with LB chickens (Fig. 3A). Among them, the most interesting candidate DEGs to be implicated in follicle selection and differentiation, including *GTSF1*, *P2RX1*, *GABRA1*, *GABRB2*, *NGF*, *ADGRD1*, *ADCY7*, *P2RX7*, *SRGN*, *SOX9*, *BCL2A1*, *GPR65*, *BCL2L14*, *ALDH1A1*, *BLK*, *GABRG1*, *METRN*, *P2RY1*2, *CYP1C1*, *ADRB2*, *TENM*2, *GPR15*7, *NDUFAB1*, *STMN3*, *PRLL*, *SOCS2*, *TLE4Z1*, *NCAM2*, *DTHD1*, *LOC424014*, *CYP2AB3*, and *HSD17B1* were found (Table 3). Furthermore, the significant top 20 signaling pathways were identified (Fig. 3B). Among them, only two of the pathways, NF-kappa B signaling pathway, and CAMs, may be much closely linked with follicle development.

**Table 3:** Characterization of partial DEGs in SYF follicles of JB and LB chickens

Name	Description	Regulation	Chr.	Log_2_FC	*P*-value
*GTSF1*	gametocyte specific factor 1	up	27	1.0872	0.0009
*P2RX1*	purinergic receptor P2X 1	up	19	1.7095	0.0383
*GABRA1*	gamma-aminobutyric acid type A receptor alpha 1 subunit	up	13	1.7586	1.2116E-05
*GABRB2*	gamma-aminobutyric acid type A receptor beta2 subunit	up	13	1.5317	0.0028
*NGF*	nerve growth factor	up	26	1.7033	0.0481
*ADGRD1*	adhesion G protein-coupled receptor D1	up	15	2.6266	0.0187
*ADCY7*	adenylate cyclase 7	up	11	1.0016	0.0289
*P2RX7*	purinergic receptor P2X 7	up	15	1.6691	4.0602E-05
*SRGN*	serglycin	up	6	1.9189	0.0205
*SOX9*	SRY (sex determining region Y)-box 9	up	18	1.5868	0.0084
*BCL2A1*	BCL2 related protein A1	up	10	1.6446	0.0340
*GPR65*	G protein-coupled receptor 65	up	5	1.3260	0.0187
*BCL2L14*	BCL2 like 14	up	1	4.3212	1.7558E-05
*ALDH1A1*	aldehyde dehydrogenase 1 family member A1	up	Z	2.3347	0.0099
*BLK*	BLK proto-oncogene, Src family tyrosine kinase	up	3	1.3837	0.0034
*GABRG1*	gamma-aminobutyric acid type A receptor gamma1subunit	up	4	1.8060	0.0005
*METRN*	meteorin, glial cell differentiation regulator	up	14	1.0096	0.0087
*P2RY1*2	purinergic receptor P2Y12	up	9	1.5714	0.0255
*LOC424014*	putative methyltransferase DDB_G0268948	up	7	2.0292E-06	2.3334
*CYP1C1*	cytochrome P450 family 1 subfamily C polypeptide 1	up	5	2.8918	0.0053
*ADRB2*	adrenoceptor beta 2	up	13	1.2618	0.0388
*TENM*2	teneurin transmembrane protein 2	down	13	−1.4428	1.0434E-05
*GPR15*7	G protein-coupled receptor 157	down	21	−1.14768	0.0104
*NDUFAB1*	NADH: ubiquinone oxidoreductase subunit AB1	down	14	−1.0438	0.0180
*STMN3*	stathmin 3	down	20	−1.5957	0.0110
*PRLL*	prolactin-like protein	down	1	−1.5757	0.0130
*SOCS2*	suppressor of cytokine signaling 2	down	1	−1.2871	0.0062
*TLE4Z1*	transducin like enhancer of split 4-Z1	down	Z	−1.0419	0.0164
*NCAM2*	neural cell adhesion molecule 2	down	1	−6.8712	0.0013
*DTHD1*	death domain containing 1	down	4	−2.1469	3.5106E-05
*CYP2AB3*	cytochrome P450, family 2, subfamily AB, polypeptide 3	down	9	−1.6150	0.0142
*HSD17B1*	Hydroxyl steroid (17-beta) dehydrogenase 1	down	27	−1.6963	0.0243

**Fig. 4:**
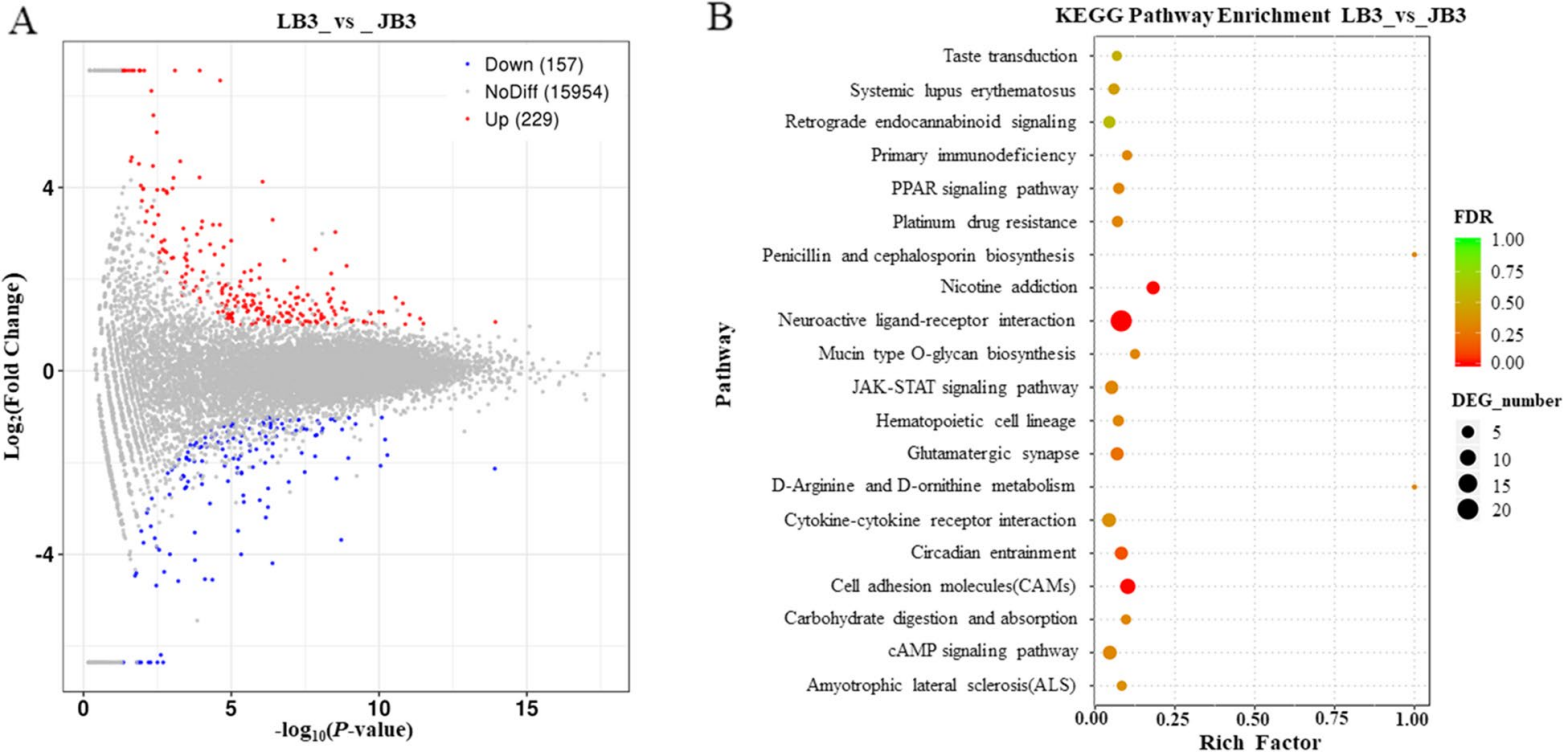
Scatter plot of annotated differently expressed genes and enriched signaling pathways in LYF follicles between JB and LB chickens. **A** MA plot of differently expressed genes in GWF follicles between JB and LB samples. JB3, LYF follicle samples of JB hens; LB3, LYF samples of LB hens. **B** Bubble chart of top 20 of KEGG pathway enrichment

In LYF follicles, 229 of up-regulated and 157 of down-regulated DEGs were mined from JB and LB chickens (Fig. 4A). Of which, the most promising DEGs to be involved in follicle growth, differentiation and maturation such as *APCDD1*, *GABRA1*, *TSHB*, *APOBEC2*, *PRLR*, *GHRHR-LR*, *NPY4R*, *BMPR2*, *TRH*, *HPGD*, *LEPR*, *CYP2D6*, *VIP*, *GREB1*, *SPEF2*, *PERP1*, *CRH*, *MAP3K9*, *RGS2*2, *FABP7*, *SSTR2*, *RBP3*, *NDUFAB1*, *STMN3*, *PRLL*, *GAP43*, *NCAM2*, *LOC770492*, *CDKN1A*, *ID4*, *LOC424014*, and *CYP2AC2* were unveiled (Table 4). Moreover, the significant top 20 signaling pathways were obtained (Fig. 4B), which included retrograde endocannabinoid signaling, PPAR signaling pathway, neuroactive ligand-receptor interaction, mucin type O-glycan biosynthesis, JAK-STAT signaling pathway, circadian entrainment, CAMs, and cAMP signaling pathway.

**Table 4:** Characterization of partial DEGs in LYF follicles of JB and LB chickens

Name	Description	Regulation	Chr.	Log_2_FC	*P*-value
*APCDD1*	APC down-regulated 1	up	2	1.4852	0.0052
*GABRA1*	gamma-aminobutyric acid type A receptor alpha 1 subunit	up	13	1.8192	1.0197E-06
*TSHB*	thyroid stimulating hormone beta	up	26	2.8128	0.0113
*APOBEC2*	apolipoprotein B mRNA editing enzyme catalytic subunit 2	up	26	1.0499	0.0021
*PRLR*	prolactin receptor	up	Z	2.1874	8.7121E-07
*GHRHR-LR*	growth-hormone releasing hormone-like peptide receptor	up	2	3.7088	0.0236
*NPY4R*	neuropeptide Y receptor Y4	up	6	4.0431	0.0204
*BMPR2*	bone morphogenetic protein receptor type 2	up	7	1.0301	0.0253
*TRH*	thyrotropin releasing hormone	up	12	1.6478	0.0474
*HPGD*	15-hydroxyprostaglandin dehydrogenase	up	4	1.4899	0.0168
*LEPR*	leptin receptor	up	8	1.1635	0.0002
*CYP2D6*	cytochrome P450 family 2 subfamily D member 6	up	1	1.5767	0.0030
*VIP*	vasoactive intestinal peptide	up	3	2.4115	0.0174
*GREB1*	growth regulating estrogen receptor binding 1	up	3	1.0473	0.0006
*SPEF2*	sperm flagellar 2	up	Z	1.3693	0.0018
*PERP1*	TP53 apoptosis effector	up	3	4.2193	6.2651E-06
*CRH*	corticotropin releasing hormone	up	2	4.5714	1.4447E-07
*MAP3K9*	mitogen-activated protein kinase kinase kinase 9	up	5	1.1066	0.0074
*RGS2*2	regulator of G protein signaling 22	up	2	1.1938	0.0013
*FABP7*	fatty acid binding protein 7	up	3	1.0730	0.0004
*SSTR2*	somatostatin receptor 2	down	18	−1.6523	0.0161
*RBP3*	retinol binding protein 3	down	6	−2.8177	0.0003
*NDUFAB1*	NADH: ubiquinone oxidoreductase subunit AB1	down	14	−2.2040	1.9938E-10
*STMN3*	stathmin 3	down	20	−1.2830	0.0434
*PRLL*	prolactin-like protein	down	1	−1.4488	0.0323
*GAP43*	growth associated protein 43	down	1	−2.7814	0.0468
*NCAM2*	neural cell adhesion molecule 2	down	1	−2.0656	0.0087
*LOC770492*	ras-related and estrogen-regulated growth inhibitor-like	down	5	−1.0108	0.0180
*CDKN1A*	cyclin dependent kinase inhibitor 1A	down	26	−1.0877	0.0151
*ID4*	inhibitor of DNA binding 4, HLH protein	down	2	−1.2727	6.0076E-05
*LOC424014*	putative methyltransferase DDB_G0268948	down	7	−1.5789	6.3016E-05
*CYP2AC2*	cytochrome P450, family 2, subfamily AC, polypeptide 2	down	3	−3.8372	0.0022

To reveal the most important DEGs and signaling pathways potentially involved in follicle development and differentiation across the three developmental stages GWF, SYF and LYF, the *NDUFAB1* and *GABRA1* genes, two most promising candidates potentially associated with egg-laying performance were screened out from the 13 co-expressed DEGs in the GWF, SYF and LYF samples. The three crucial signaling pathways, including PPAR signaling pathway (ko 03320), cAMP signaling pathway (ko 04024), and neuroactive ligand-receptor interaction (ko 04080) were significantly enriched. Moreover, *NDUFAB1* gene as a member of oxidative phosphorylation pathway (gga: 00190; Supplementary Fig. S1) was involved in the PPAR signaling pathway; while *GABRA1* gene as a component of neuroactive ligand-receptor interaction pathway (gga: 04080; Supplementary Fig. S2) was implicated in the G protein-coupled receptor pathway (gga: 04030) which contains the FSH/FSHR signaling pathway, and in the cAMP signaling pathway.

### Validation of the selected DEGs by RT-qPCR

**Fig. 5:**
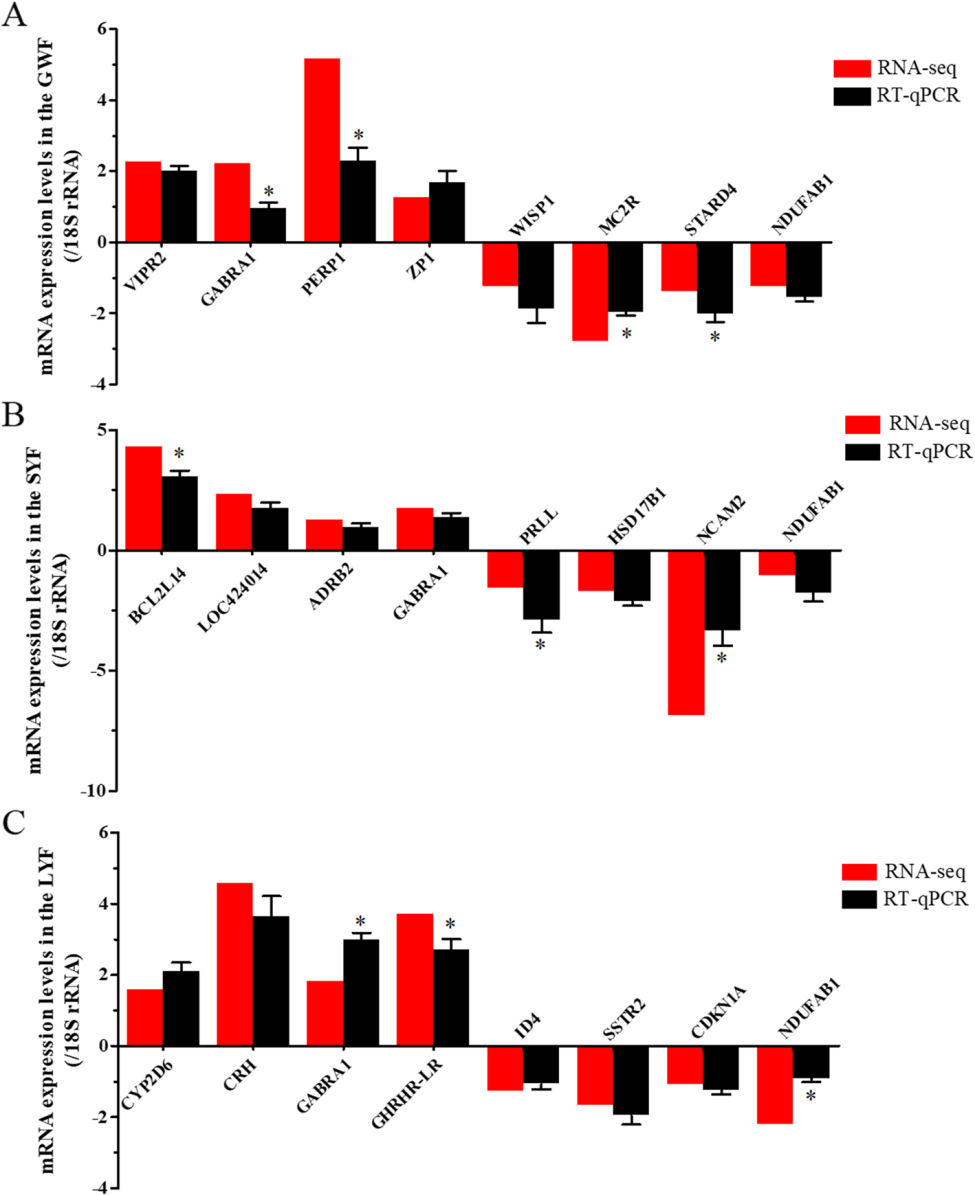
Evaluation and comparison of mRNA expression levels (Log_2_FC) of 20 candidate genes when analyzed by RNA Seq and quantitative RT-qPCR. **A** The candidates in the differentially expressed in GWF follicles between JB and LB hens. **B** The candidates in SYF follicles. **C** The candidates in LYF follicles. The relative expression level of each gene was quantified by the 2^−ΔΔCT^ method and *18S rRNA* was used as the internal control for normalization of the results

Based on the analyses aforementioned, 20 representatives of most relevant DGEs, i.e., *VIPR2*, *GABRA1*, *PERP1*, *ZP1*, *WISP1*, *MC2R*, *STARD4* and *NDUFAB1* that differentially expressed in GWF follicles, *BCL2L14*, *LOC424014*, *ADRB2*, *GABRA1*, *PRLL*, *HSD17B1*, *NCAM2* and *NDUFAB1* in SYF follicles, *CYP2D6*, *CRH*, *GABRA1*, *GHRHR-LR*, *ID4*, *SSTR2*, *CDKN1A* and *NDUFAB1* in LYF follicles between JB and LB hens were selected for validation by RT-qPCR. The results showed the significantly differential expression levels of the most transcripts determined by RT-qPCR analysis are consonant with the observation detected by RNA-seq (Fig. 5). While there are significant differences in the expression levels of the other genes, i.e., *GABRA1*, *PERP1*, *BCL2L14*, *GHRHR-LR*, *MC2R*, *STARD4*, *NDUFAB1*, *PRLL*, and *NCAM2*, the observed expression trends were concordant with the RNA-seq results, confirming the transcriptome sequencing data are reliable, and can be further investigated for understanding the genetic mechanism of ovarian follicle development associated with egg production in chicken.

### Confirmation of the roles of *NDUFAB1* and *GABRA1* genes in ovarian follicle development

**Fig. 6:**
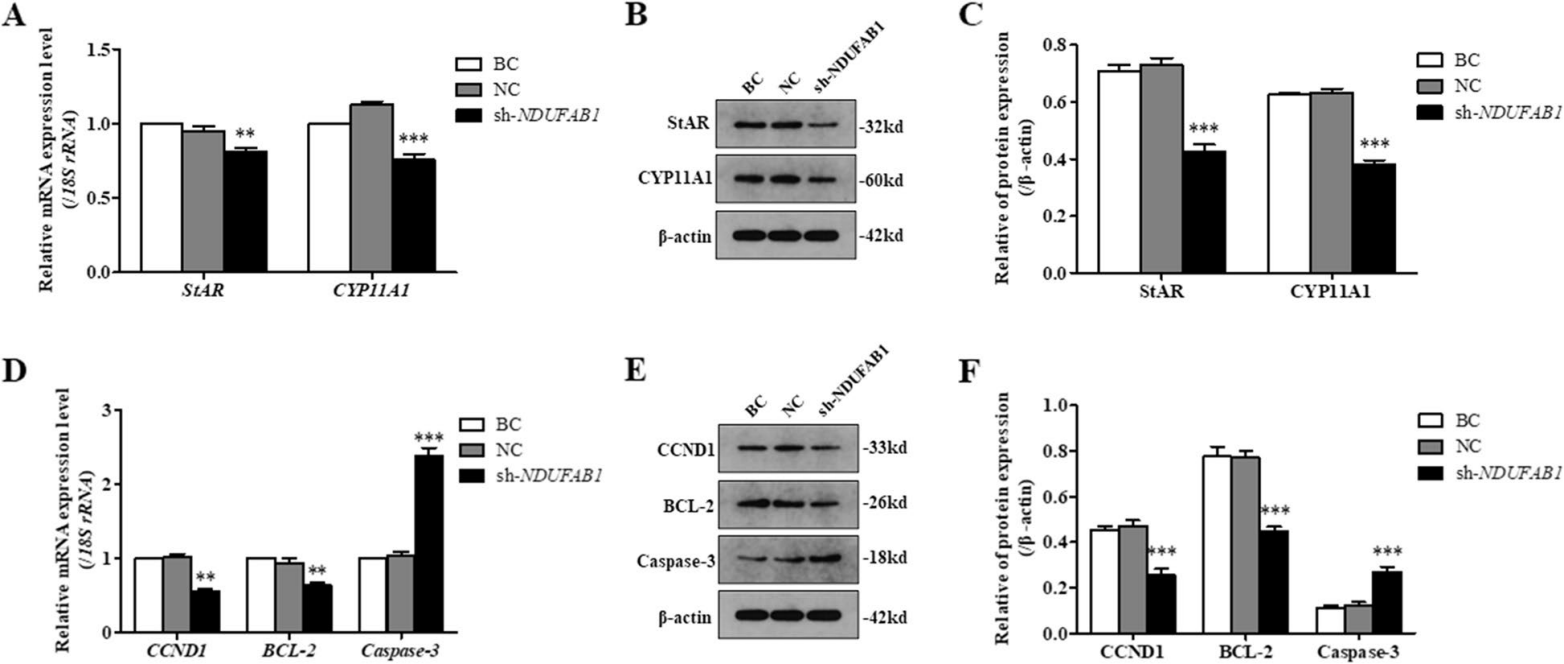
Silence of NDUFAB1 in the GCs. sh-*NDUFAB1*, GCs being transfected with *NDUFAB1*-specific shRNA; NC, scrambled shRNA (negative control); BC, no shRNA as a vehicle (blank control). **A** The *STAR* and *CYP11A1* mRNA expression in the GCs was analyzed using RT-qPCR. **B**, **C** Expression of STAR and CYP11A1 proteins in the GCs with or without interference using the shRNA was analyzed by western blotting; β-actin was used as a loading control. **D** The *CCND1*, *BCL-2* and *caspase-3 (CASP3)* mRNA expression in the GCs was analyzed using RT-qPCR. **E, F **Expression of CCND1, BCL-2 and caspase-3 proteins in the GCs with or without interference using the shRNA was analyzed by western blotting

**Fig. 7:**
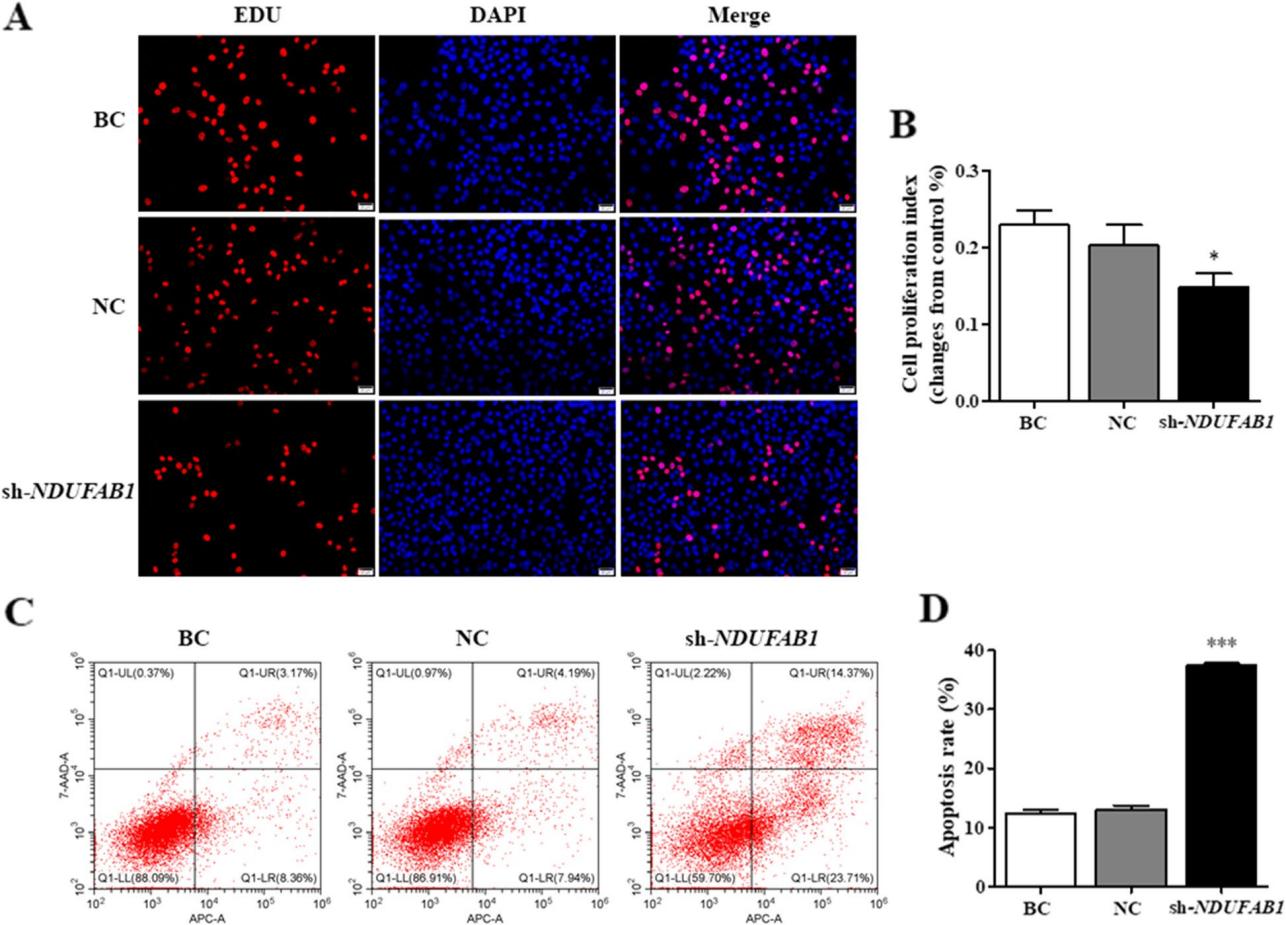
Effects of NDUFAB1 silence on the GC proliferation and apoptosis. sh-*NDUFAB1*, GCs were transfected with *NDUFAB1*-specific shRNA; NC, scrambled shRNA; BC, no shRNA as a vehicle. **A**, **B** NDUFAB1 silence on the GC proliferation. **C**, **D** NDUFAB1 silence on cell apoptosis. Total number of apoptotic cells comprises the number of cells in the right upper quadrant (Q1-UR) and number of cells in the right lower quadrant (Q1-LR)

To further confirm the exact roles of *NDUFAB1* and *GABRA1* genes in ovarian follicle development, silence of the genes was performed by infection of the shRNA lentivirus in the GCs from 6 to 8 mm follicles of hen ovaries, respectively. The result demonstrated that in the *NDUFAB1* silencing GCs, expression levels of STAR, CYP11A1, CCND1, and BCL-2 proteins as well as all of their transcripts were significantly downregulated (*P* < 0.01, Fig. 6; Supplementary Fig. S3), but the expression Caspase-3 and its transcript were remarkably upregulated (*P* < 0.001). Simultaneously, cell proliferation ratio of the GCs was notably decreased as compared to the negative control (*P* < 0.05, Fig. 7), whereas cell apoptosis rate was markedly increased (*P* < 0.001). It was suggested that *NDUFAB1* promotes follicle development by stimulating GC proliferation and decreasing cell apoptosis, increases the expression of CCND1 and BCL-2 but attenuates the expression of caspase-3, and facilitates steroidogenesis by enhancing the expression of STAR and CYP11A1.

**Fig. 8:**
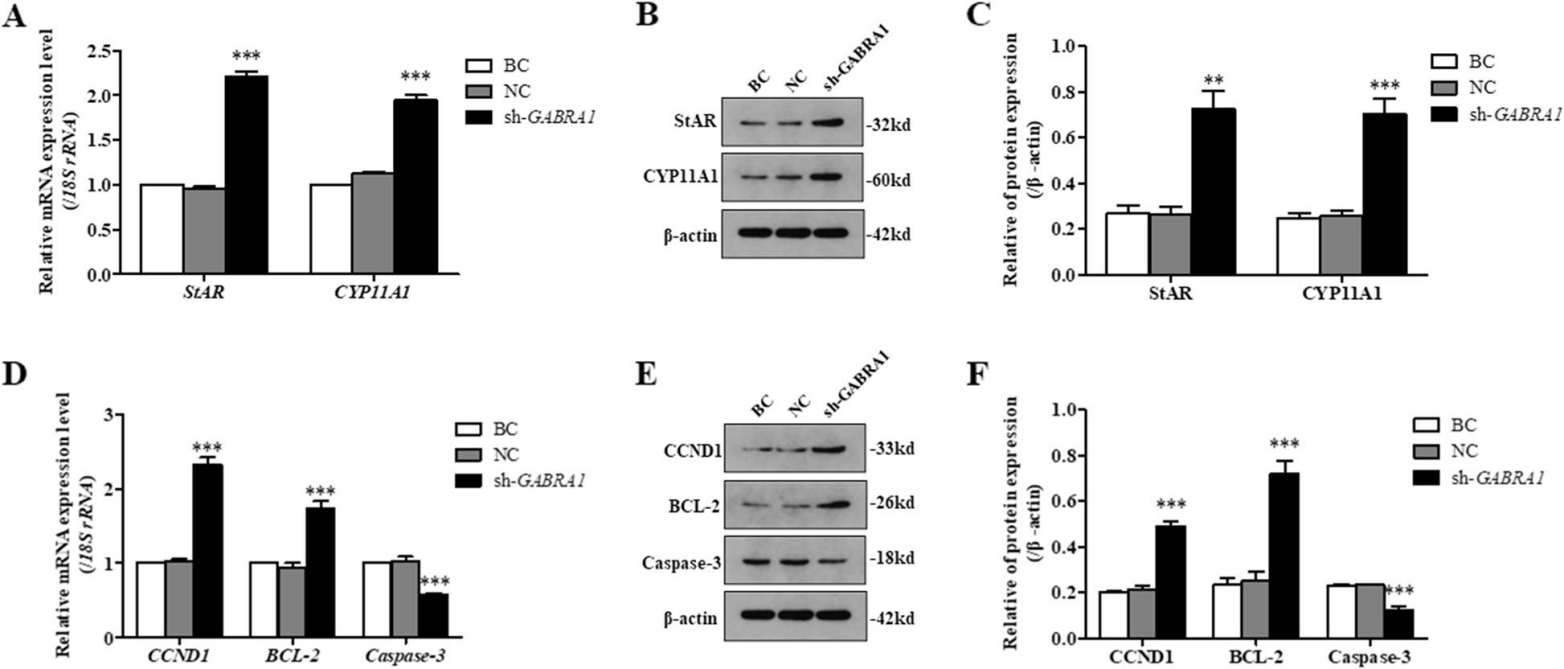
Silence of GABRA1 in the GCs. sh-*GABRA1*, GCs being transfected with *GABRA1*-specific shRNA; NC, scrambled shRNA; BC, no shRNA as a vehicle. **A** The *STAR* and *CYP11A1* mRNA expression in the GCs was analyzed using RT-qPCR. **B**, **C** Expression of STAR and CYP11A1 proteins in the GCs with or without interference using the shRNA was analyzed by western blotting; β-actin was used as a loading control. **D** The *CCND1*, *BCL-2* and *CASP3* mRNA expression in the GCs was analyzed using RT-qPCR. **E**, **F** Expression of CCND1, BCL-2 and caspase-3 proteins in the GCs with or without interference using the shRNA was analyzed by western blotting

**Fig. 9:**
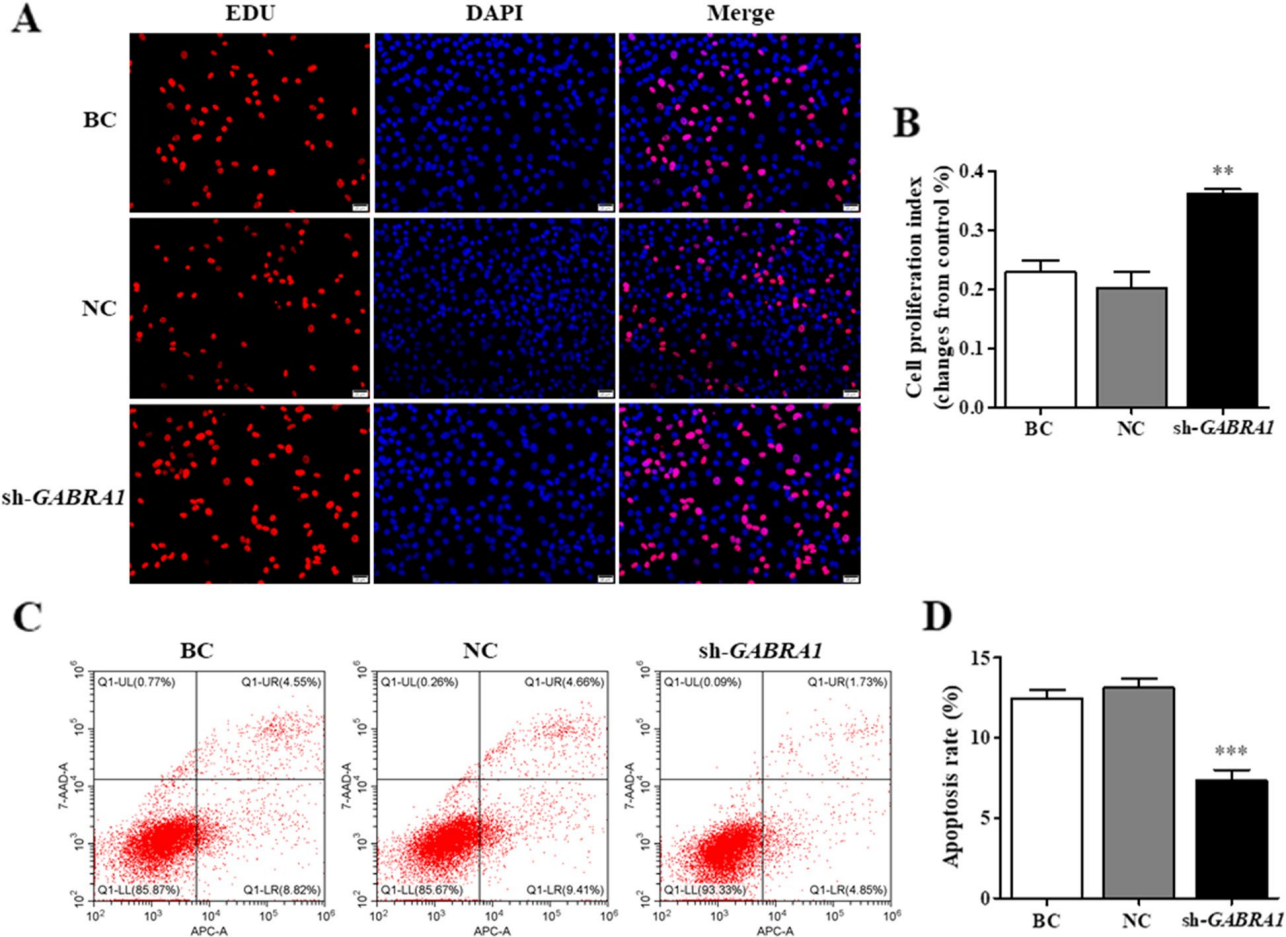
Effects of GABRA1 silence on the GC proliferation and apoptosis. sh-*GABRA1*, GCs were transfected with *GABRA1*-specific shRNA; NC, scrambled shRNA; BC, no shRNA as a vehicle. **A**, **B** GABRA1 silence on the GC proliferation. **C**, **D** GABRA1 silence on cell apoptosis

For *GABRA1* silence of GCs, the expression levels of *STAR*, *CYP11A1*, *CCND1*, and *BCL-2* transcripts and their proteins were notably elevated (*P* < 0.01, Fig. 8; Supplementary Fig. S4). Oppositely, the expression *CASP3* transcript and its protein were significantly reduced (*P* < 0.001). Furthermore, cell proliferation ratio of the GCs was remarkably enhanced as compared to the negative control (*P* < 0.01, Fig. 9), conversely, cell apoptosis rate was sharply decreased (*P* < 0.001). It was proposed that *GABRA1* inhibits GC proliferation and increases cell apoptosis, decreases the expression of CCND1, BCL-2, STAR, and CYP11A1 but elevates the expression of caspase-3.

## Discussion

Ovary is an important reproductive organ in adult poultry, which contains many various-sized follicles corresponding to the different follicular developmental stages, including the small white follicle resting (months or years) stage (< 2 mm), the slow growing undifferentiated prehierarchical stage (white, 2–6 mm), the stage of follicle selection (small, yellow; 6–8 mm), and the final differentiated hierarchy (large, yellow) stage [1, 4, 5]. The follicles mainly composed of oocytes, granulosa cell layers (GCs) and theca cell layers, are the major compartments which enable the ovary to execute its dual function of gametogenesis and steroidogenesis in chicken [25]; its development is a highly intricate and coordinated hierarchical process involving a multitude of biological events controlled by reproductive hormones in the ovary [8, 26, 27]. Undoubtedly, the follicles at the various developmental stages possess their own distinct molecular genetic characteristics and play different roles in contributing to ovary growth and development. Particularly, genetic regulation of 6–8 mm diameter follicles is generally involved in follicle selection [2, 28, 29] and may possess an exclusive influence on hierarchy of undifferentiated prehierarchical follicles. However, the major genes controlling the follicle development at each stage and their exact physiological mechanisms that regulate follicular growth and order of the ovarian follicle hierarchy remain largely unknown.

To mine the key genes implicated in follicular development at the stages of follicle selection, before and after the selection, in this study, transcriptome analysis of the GWF, SYF and LYF follicles was implemented between the JB and LB chicken breeds. To our knowledge, this is one of the few studies to reveal potential pivotal genes of involvement in hen ovarian follicles at the developmental stages immediately before and after follicle selection, which may be associated with high and low rates of egg production. Firstly, in the GWF follicles of this study, the JB hens with low egg production showed higher mRNA levels of *VIPR2*, *GABRA1*, *PERP1*, and *ZP1*, and lower mRNA levels of *WISP1*, *MC2R*, *STARD4*, and *NDUFAB1* genes, in which the most representative gene *VIPR2* is also named pituitary adenylate cyclase-activating polypeptide (PACAP) receptor (*VPAC2*), encoding the VPAC2/vasoactive intestinal peptide (VIP) receptor (VIPR2) belonging to the VIP/PACAP type II receptors, including VPAC1/VIPR1 and VPAC2/VIPR2 [30, 31]. Previous studies have proved that PACAP, a bioactive peptide transiently expressed in preovulatory follicles, stimulates various ovarian functions, such as intracellular cyclic AMP (cAMP) accumulation, plasminogen activator production, and inhibits GC apoptosis by interacting with its receptors, PACAP type I receptor (PAC1) and the type II receptors in rat [32–34]. Moreover, PACAP stimulates oocyte growth but block its maturation in early follicles by acting directly on the oocyte via PAC1 and VPAC2 receptors, whose expression is dominant in growth phase [35]. The participation of VIP in ovarian steroids production was confirmed to induce polycystic ovary [36, 37]. Therefore, it was proposed that the higher expression of *VIPR2* transcript in GWF developmental stage may be relevant to broodiness and lower egg-laying trait of JB breed by blocking follicle growth and maturation in ovarian follicle development.

In this study, transcriptomic analysis of SYF follicles revealed higher mRNA levels of *BCL2L14*, *LOC424014*, *ADRB2*, and *GABRA1*, and lower mRNA levels of *PRLL*, *HSD17B1*, *NCAM2*, and *NDUFAB1* genes in the JB hens as compared with LB hens, in which the most representative genes, *ADRB2* and *HSD17B1*, have been intensively studied in ovarian functions [38, 39]. The β2 adrenergic receptor (ADRB2) encoded by *ADRB2* gene was mainly expressed in the GCs and Theca cells (TCs) of ovarian follicles and regulated the levels of cAMP and steroid production through activation of ADRB2/cAMP/protein kinase A (PKA) signaling pathway and/or ADRB2/cAMP/protein, phospholipase C (PLC)/protein kinase C (PKC)/cAMP response element-binding protein (CREB) signaling cascade [40–42]. However, the excessive ovarian steroidal response to gonadotropins and beta-adrenergic stimulation enhanced polycystic ovary syndrome (PCOS), an endocrine disorder characterized by anovulation, hyperandrogenism and polycystic ovaries [36, 37, 43, 44]. The much abundant expression levels of *ADRB2* gene may induce layer broodiness by activation of adenylate cyclase through the action of G proteins and stimulate anovulation [37, 43].

The hydroxysteroid (17beta) dehydrogenase type 1 (HSD17B1) is a steroidogenic enzyme encoded by *HSD17B1* gene, to efficiently catalyze reversible interconversion of a low-active precursor estrogen estrone (E1) to the highly active E2 that is necessary for normal ovary development [13, 45]. It is the major isozyme in the granulosa cells of the ovary and has a central role in regulating the circulating estradiol concentration as well as its local production in estrogen target cells, locally promotes development, differentiation, and maturation of the follicle [46–48]. Nevertheless, inhibition of HSD17B1 impairs the synthesis of 17β-estradiol, and attenuates action of the estradiol [47, 49], which can directly block ovarian follicle development. Furthermore, HSD17B1 plays a crucial role in controlling cell proliferation and in the regulation of the growth and function of organs [50]. It was suggested that the lower expression levels of *HSD17B1* transcript in SYF follicles of JB hens may affect ovarian dominant follicle selection and follicle growth and function by repressing 17β-estradiol production and follicle cell proliferation, and finally lead to a low egg production.

Transcriptomic analysis of LYF follicles revealed higher mRNA levels of *CYP2D6*, *CRH*, *GABRA1*, and *GHRHR-LR*, and lower mRNA levels of *ID4*, *SSTR2*, *CDKN1A* and *NDUFAB1* genes in the JB than in the LB layers. Among them, the most representative gene *GHRHR-LR*, also named *VIPR1*, its encoding product VIPR1 was mainly expressed in granulosa cells and residual ovarian tissue [51]. PACAP may promote oocyte maturation in the maturation phase via VPAC1-R on the follicle cells, whose expression surges in full-grown follicles prior to maturation and is consistently high in the follicles undergoing final maturation [35]. Moreover, the genetic polymorphisms of *VIP* and *VIPR1* genes were associated with chicken broodiness and egg production [52, 53]. It was intimated that the higher expression levels of *VIPR1* transcript in LYF follicles of JB hens may inhibit ovarian follicle growth, differentiation and maturation, and contribute to the lower egg production.

Interestingly, the significantly up-regulated *GABRA1* mRNA and down-regulated *NDUFAB1* mRNA of the GWF, SYF and LYF follicles were co-expressed differentially in JB hen ovaries when compared with LB hen. Previous studies have reported that *GABRA1* gene encodes γ-aminobutyric acid type A receptor α1 subunit (GABRA1) is one subtype of the neurotransmitter γ-aminobutyric acid (GABA) activating GABA_A_ receptors that act as the components of the gamma-aminobutyric acid signaling pathway [54, 55]. The heteropentameric receptors are formed from an array of subunits (α1–6, β1–3, γ1–3, δ, ε, π, θ and ρ1–3), producing receptors with diverse functional and pharmacological properties [56–58]. The GABA_A_ receptors are vital for controlling neuronal excitability and can display significant levels of constitutive activity that contributes to tonic inhibition [58]. It has been established that the progesterone metabolite allopregnanolone, a potent modulator of the GABA_A_ receptor, is sedative in high concentrations but may precipitate paradoxical adverse effects on mood at levels corresponding to luteal phase concentrations in susceptible women [59]. It indicated that GABA_A_ receptors may be loosely linked to reproductive performance in chicken. Furthermore, And the configuration of GABRA1 proves the receptor’s vital role in apoptosis regulations, mitochondrial function, inhibitory effects of different neurotransmitters, and synaptic plasticity by interaction with neuroactive steroids such as androstane and progesterone derivatives [57, 60]. Moreover, another studies on *NDUFAB1* gene demonstrated that the *NDUFAB1* gene encodes the acyl carrier protein (ACP), NDUFAB1 subunit of mitochondrial complex I (NADH: ubiquinone oxidoreductase), which is involved in cytosolic and mitochondrial fatty acid synthesis pathways [61]. ACP molecule is a universal and highly conserved carrier of acyl intermediates during fatty acid synthesis [62], exists either in the cytosol as a separate domain within a large multifunctional fatty acid synthase polyprotein (type I FAS) or in mitochondria as a discrete entity ACP of the bacterial type II FAS [63, 64]. However, the exact roles of *GABRA1* and the *NDUFAB1* in ovarian follicular function remain unclear in chicken.

To further confirm the roles of *GABRA1* and *NDUFAB1* genes in ovarian follicle development, silencing *GABRA1* and *NDUFAB1* genes were conducted in the GCs of SYF, respectively. The result showed that expression levels of the steroidogenic-related genes, both *STAR* and *CYP11A1*, were remarkably enhanced when silencing *GABRA1* was implemented in the GCs. As known, the STAR and P450scc were two key enzymes in regulation of P4 synthesis and secretion within the GCs. In which, STAR functions to transport cholesterol to the inner membrane of mitochondria from the outer membrane [65], then P450scc catalyzes transformation of cholesterol to pregnenolone, a P4 precursor [66]. It is well shown that as follicle was recruited into the follicular hierarchy (follicle selection), the granulosa cells initiate differentiation, and accompanied by the P4 synthesis and secretion [67, 68]. P4 is required for growth and ovulation of preovulatory follicles, exerts a stage-dependent role in ovarian follicle development [69]. However, high level of progesterone markedly inhibited ovulation of preovulatory follicles, and generally delayed oviposition by negative feedback fashion on the gonadotroph in hens [69, 70]. In this study, the higher expressions of GABRA1 can decrease P4 production by inhibiting *STAR* and *CYP11A1* expression level. But the much lower P4 level or P4 absence also suppressed folliculogenesis and ovulation [71, 72]. Concurrently, expression levels of cell proliferation related genes *BCL-2* and *CCND1* genes were significantly elevated in the GCs, whereas the expression of *CASP3* gene was notably decreased. Furthermore, the cell proliferation ratio of the GCs was remarkably enhanced as compared to the negative control; conversely, cell apoptosis rate was sharply decreased as *GABRA1* expression was silenced. Although the exact roles and regulatory mechanism of GABRA1 and the GABAR in ovarian follicular function remain unclear in chicken, a study reported the inhibitory effect of xanthohumol on evoked glutamate release was antagonized by suppressing the GABAR/Ca^2+^-calmodulin/AC/cAMP/PKA cascade in rat hippocampus [73]. Moreover, the AC/cAMP/PKA pathway was involved in granulosa cell proliferation and differentiation of ovarian follicles in hen [74, 75]. Our result showed *GABRA1* gene plays an inhibitory role in ovarian follicle development, follicle selection and differentiation by suppressing steroidogenesis, GC proliferation and stimulating cell apoptosis of SYF.

As aforementioned, it was found that the expression *NDUFAB1* mRNA in the ovarian follicles of three developmental stages was down-regulated in the JB hens. Previous studies corroborated that NDUFAB1 is linked directly to mitochondrial ATP production, indirectly to potential reactive oxygen species generation [76], and reduced mitochondrial ATP production directly resulted in increased cell apoptosis [77]. Moreover, down-regulation of mitochondrial ACP expression in HEK293T cells has brought about a slowing of cell growth rate or a high level of cell death [78]. It was consistent with our results that the GC proliferation ratio was notably decreased; moreover, cell apoptosis rate was markedly increased when *NDUFAB1* expression was silenced. Simultaneously, it was accompanied by the significant downregulation of STAR, CYP11A1, CCND1, and BCL-2 expression, and remarkably upregulated Caspase-3 expression. It confirmed that NDUFAB1 has a positive regulation in ovary follicular growth and differentiation at the developmental stage of follicle selection by enhancing steroidogenesis, GC proliferation and decreasing cell apoptosis. Furthermore, this study has shown the expression levels of *NDUFAB1* mRNA across the three developmental stages of GWF, SYF and LYF in ovary development keeps lower in JB hens than in LB layers, but the higher expression of *GABRA1* transcripts. Accordingly, it was concluded both *NDUFAB1* and *GABRA1* genes were strongly associated with egg-laying production of hens.

Additionally, in this study three crucial signaling pathways including PPAR signaling pathway, cAMP signaling pathway, and neuroactive ligand-receptor interaction were screened out. Among them, the cAMP signaling pathway was not only involved in the neuroactive ligand-receptor interaction such as the GABAR/Ca^2+^-calmodulin/AC/cAMP/PKA cascade of the GABAergic synapse pathway [54, 73], it also provokes a variety of processes required for ovarian follicle growth, selection, differentiation, and maturation [74, 79]. As is well known, FSH is essential for reproductive system including steroidogenesis, folliculogenesis, and follicular maturation by interaction with FSH receptor (FSHR) in an endocrine dependent manner [80, 81]. FSHR, a member of the superfamily of G-protein-coupled receptors, is exclusively expressed on granulosa cells of ovarian follicles and mediates FSH signal transduction through cAMP signaling pathway [2, 28, 82]. The PPAR signaling pathway was previously reported to affect ovarian follicle development and normal ovarian function by being indirectly involved in oocyte maturation and ovulation via regulation of steroid hormone synthesis in granulosa cell [74]. Accordingly, the key candidate genes such as *NDUFAB1*, *MC2R*, *VIPR2*, *GHRHR-LR*, *MC2R* and *SSTR2* that were identified currently may be implicated in granulosa cell proliferation and apoptosis, oocyte meiosis and maturation, follicular differentiation and atresia, and secretion of gonadotrophin-release hormone via crosstalk or intracellular interactions with the PPAR pathway [61, 79, 83–86].

## Conclusions

Collectively, the transcriptome comparative analysis of ovarian follicles at the GWF, SYF and LYF developmental stages reveals the key genes and signaling pathways involved in ovarian follicular follicle growth (including follicle selection), differentiation, and maturation, which has provided molecular evidences for new insight into the regulatory mechanism underlying ovarian follicle development associated with egg production in chicken.

## Methods

### Chicken raising management and trait measurement

After hatching, LB and JB hens were raised in layered batteries under the same rearing conditions, including free access to water and feed in accordance with the recommendations for nutrient levels of in Lohmann breeds [24, 87]. Approaching 16 weeks of age, hens were reared in individual cages under constantly maintained conditions. All of the layers were exposed to a 16 L: 8D photoperiod, with lights on at 5:00 AM. Age at first egg was recorded after the start of laying and egg production was observed daily, with egg weights determined on 1 day per week. Following feed and water restrictions at 21 and 66 weeks of age, BW was recorded and the individual laying performance calculated. Egg-laying traits examined in this study included hen-housed egg production (egg-laying number) at 21, 30, 43, 57, and 66 weeks of age. Average egg production rates, average egg weight and body weight of LB and JB hens at the ages were calculated and compared.

### Samples preparation and cell culture

In this study, ten layers of every JB and LB breeds were obtained and euthanized at 21 weeks of age, and the GWF, SYF and LWF follicles were harvested. After the surrounding vascular and connective tissues of the follicles were removed with fine forceps and a scalpel [88], follicles were immediately snapped frozen in liquid nitrogen and preserved at − 80 °C for RNA extraction. All experiments and methods were performed in accordance with the ARRIVE guidelines.

The GCs from the SYFs were isolated and cultured in Medium199 (M199; Gibco, Waltham, MA, USA) supplemented with 10% (v/v) fetal calf serum (Gibco) at 37 °C with 5% CO_2_ in humidified chambers following our published method [8, 89]. The cultured GCs used in this experiment have been purified and quantified in our laboratory. The specificity of the GCs has been identified by the H & E staining procedure and fluorescence staining analysis [25, 90].

Total RNA was isolated from follicles of each hen using Trizol Reagent (Invitrogen Life Technologies, Carlsbad, CA, USA) according to the recommended manufacturer’s protocol, and concentration, quality, and integrity were determined by a Thermo NanoDrop 8000 Spectrophotometer (Thermo Fisher Scientific, Wilmington, Delaware, USA). Three micrograms of RNA were used as input material for RNA sample preparations. The other parts of the samples were stored at − 80 °C for validation experiments.

### RNA-Seq analysis

The 18 cDNA libraries were generated using the TruSeq RNA Sample Preparation Kit (Illumina, San Diego, CA, USA) according to the method published by our group [23]. Among them, nine cDNA libraries corresponding to the GWF (named A11, A12, A13), SYF (named A21, A22, A23) and LYF (named A31, A32, A33) samples from LB hens and nine cDNA libraries corresponding to the GWF (named B11, B12, B13), SYF (named B21, B22, B23) and LYF (named B31, B32, B33) samples from JB layers were constructed, respectively. Effective concentration of libraries were greater than 2 nM and libraries were qualified, sequenced, and paired-end sequencing with the 150 bp sequencing read length was performed. The cDNA libraries were sequenced on a Hiseq platform (Illumina, Inc., San Diego, CA, USA) by Shanghai Personal Biotechnology Cp. Ltd. Follow-up analyses were based on clean data with high quality. All of the clean reads were aligned with the reference genome (ftp://ftp.ensembl.org/pub/release-86/fasta/gallusgallus/dna/Gallusgallus.Gallusgallus-5.0.dna.toplevel.fa.gz) by using sequence alignment program HISAT 2.1.0 [91, 92].

### Identification of differentially expressed genes

In accordance with our earlier reported method [23], the HTSeq (ver. 0.6.1) software was utilized to count the reads mapped to each gene. The transcription level was normalized according to its FPKM (Fragments per Kilobase of transcript per Million mapped reads) values using the Cufflinks package (v2.1.1). And based upon the normalized FPKM + 1 value, expression pattern assessment for differentially expressed genes (DEGs) between case group and control group was fulfilled by using the Multi-Experiment Viewer (version 4.9.0) software (https://sourceforge.net/projects/mev-tm4/files/latest/download). The resulting *p*-values were adjusted using the Benjamini and Hochberg’s approach for controlling the false discovery rate and an absolute value of the |log_2_FoldChange| (Log_2_FC) was served as the threshold for judging significance of the gene expression. Candidate genes with an adjusted *p*-value (padj) < 0.05 and Log_2_FC > 1 were identified as differentially expressed. MA plots to visualize up- and down-regulated genes of each sample were generated by using the R package “ggplot2” (version 3.5.0), according to the values of padj and Log_2_FC [93].

### GO annotation and KEGG enrichment analysis for DEGs

An online biological tool, Gene Ontology (GO, http://geneontology.org/), was utilized to annotate and analyze the molecular and functional characteristics of the DEGs [94]. The calculated *p*-value was Bonferroni corrected, taking the corrected *p*-value < 0.05 as a threshold for GO annotation. Another online biological tool, Kyoto Encyclopedia of Genes and Genomes (KEGG, http://www.kegg.jp/), provided the comprehensive database resources for the KEGG pathway enrichment of the DEGs. In this step, four databases were utilized to reveal high-level functions and biological systems of the DEGs, including Reactome (http://www.reactome.org), KEGG pathway (http://www.genome.jp/kegg/). Results with *P* < 0.05 were considered significantly enriched by DEG.

### Data validation by quantitative real-time RT-PCR

To verify the accuracy and repeatability of the RNA-Seq results of DEGs, transcription levels of 24 representative genes in the ovarian follicles were estimated by using quantitative real-time reverse transcriptase PCR (RT-qPCR) as described previously [8]. The primers utilized for amplification of the candidate genes including *VIPR2, GABRA1*, *PERP1*, *ZP1,* and *WISP12*, et al., were listed in Table 5. Using the 2^-ΔΔCt^ method, mRNA expression results were normalized against *18S rRNA* as internal control. To quantify mRNA expression levels by RT-qPCR analysis, four amplified products in independent reactions per bird were utilized. All the experiments were carried out in triplicate using different batches of sampled follicles.

**Table 5:** Primer pairs designed for quantitative real-time PCR analysis

Gene	Forward primer (5′ - 3′)	Reverse primer (5′ - 3′)	Accession No.	Size
*VIPR2*	ATAATGACTATGAGGACGAT	TGGATGTAGTTCCGAGTA	NM_001014970	154 bp
*GABRA1*	TGTGTTTTCTGCCCTCATC	ATCCTTCACCTTCTTTGGC	NM_204318	109 bp
*PERP1*	AGACCTTGCCCTATGTGC	GAAGTTGAACCGAAGTGTAT	NM_001277703	206 bp
*ZP1*	CTCCACCATTGATGTCCAGC	TCGGCGTCAGGGTAGTAGG	NM_204683	222 bp
*WISP1*	CCAGGATTTCCAACGACA	GACAGCCAGGCACTTCTT	NM_001024579	122 bp
*MC2R*	TCTTCTACGCTTTGCGGTAC	ACTGGTTGGCAGTGAGGC	NM_001031515	245 bp
*STARD4*	AATGGACATCGTGGAAAC	AGGCTGTGGCTAGGATAG	NM_001079742	249 bp
*NDUFAB1*	AGGACGAGTTCGGCTTTG	TGACATCAGGGCCATCC	XM_004945263	157 bp
*BCL2L14*	TAAGGAACACGCAGAATC	TGTAGGAGCAGTCGCAAG	XM_040660909	231 bp
*LOC424014*	TGAGGATGGCTCGGTTGA	AATGGTGTAGGTGCTGATGG	XM_001232693	130 bp
*ADRB2*	GGAGCGACTACAACGAGG	AATCACTTCCCAAGCAACC	XM_040683024	255 bp
*PRLL*	GCAGTAGATGAAGCGATGT	GCCATACCCAACTCCCAC	NM_001165912	172 bp
*HSD17B1*	CACCGCACGCACCATTCA	CGCACTCCACCAGCGTCAT	NM_204837	206 bp
*NCAM2*	CGGCTACAAACAGAATAGGAA	ATGAATGAAACCTTGGCAGT	XM_425540	124 bp
*CYP2D6*	TTACTACAACCCGCATCT	AAGGACTTCTTTCCCATC	NM_001195557	297 bp
*CRH*	CTGGACCTGACTTTCCACCTGC	CCGCCTCACTTCCCGATGA	NM_001123031	116 bp
*GHRHR-LR*	TGGCATTCTTCCAGTTCA	GACCCAAGTAAGCATCACAG	XM_040664932	167 bp
*ID4*	ACAAGCGGGTCAGCAAAGTG	TACCGGGTCGGTGTTGAGGG	NM_204282	179 bp
*SSTR2*	CCAACTCGGAGCCAAGAC	CAAAGGGAGCAGTCAAACA	NM_001030345	164 bp
*CDKN1A*	GACCACGGAAGGGACTGA	AGACGGTGACGGACCACA	NM_204396	129 bp
*STAR*	GTCCCTCGCAGACCAAGT	TCCCTACTGTTAGCCCTGA	NM_204686	196 bp
*CYP11A1*	GCTTTGCCTTGGAGTCTGTG	TCGGTGCTCTTGCGTTGC	NM_001001756	277 bp
*CCND1*	GGAGCAGAAGTGCGAAGAGG	TGGAGTTGTCGGTGTAAATG	NM_205381	188 bp
*BCL-2*	ATGACCGAGTACCTGAACCG	CCAAGATAAGCGCCAAGAGT	NM_205339	182 bp
*CASP3*	AAGAACTTCCACCGAGATACCG	GCTTAGCAACACACAAACAAAA	NM_204725	204 bp
*18S rRNA*	ATTGGAGGGCAAGTCTGGTG	CCAGCTCGATCCCAAGATCC	AF173612	109 bp

### Small hairpin RNA (shRNA) transfection

Three small interfering (siRNA) sequences targeting *NDUFAB1* or *GABRA1* gene were designed using an InvivoGen siRNA Wizard v3.1 and the most effective siRNA was screened out as we previously reported [8, 89]. After lentiviral expression vector pLVX-shRNA2-*NDUFAB1* or -*GABRA1* carrying the specific siRNA was constructed, GCs were then transfected with the *NDUFAB1* shRNA or *GABRA1* shRNA lentivirus in 24-well plates (2 × 10^5^ cells/well), respectively; and incubated at 37 °C with 5% CO_2_. After 24 h of culture, the GCs were collected for EdU cell proliferation and cell apoptosis assay, and lysed for Western blotting and RT-qPCR analysis. The sequence information of *NDUFAB1* shRNA, *GABRA1* shRNA, shRNA negative control and the frame of lentiviral vectors was shown in Table S2. The most effective siRNA sequences were listed as below: *NDUFAB1* siRNA 5′-CCACAAGAGAUAGUAGAUUTT-3′; *GABRA1* siRNA 5′- GCAGAAUGUCCAAUGCAUUTT-3′. Scrambled siRNA was used as the negative control siRNA: 5′- UUCUCCGAACGUGUCACGUTT-3′, which was synthesized by Genepharma (Shanghai, China).

### Western blotting

Western blot analysis for STAR, CYP11A1, CCND1, BCL-2, and Caspaes-3 was performed using total cellular extracts according to our previously described [8, 89]. The affinity-purified antibodies for STAR and the other antibodies were used (Supplementary Table S3). Briefly, equal amounts of protein were separated by 10% (w/v) SDS-polyacrylamide gel under reducing conditions and electro-transferred to Protran nitrocellulose membranes (Whatman, Dassel, Germany). After electrophoresis of the protein samples in a mini gel apparatus, a pre-stained protein molecular weight marker was loaded to locate/monitor the target proteins in the electrophoresis (SDS-PAGE). At the approximated protein size position, the gel was directly cut and transferred to the nitrocellulose membrane for western blotting. The horseradish peroxidase-conjugated anti-rabbit or anti-mouse IgG secondary antibody was incubated for 2 h at room temperature. Blots were subsequently performed with ECL western blotting agent (Rockford, IL, USA) for 5 min and exposed to X-ray film for 1–5 min. The signals were detected using the ECL Plus Western blotting detection system according to the manufacturer’s instructions. Anti-β-actin (dilution 1:500, Boster, Wuhan, China) antibody acted as a loading control. As a result, the molecular size of the ladders was not observed in the original blots.

### EdU cell proliferation assay

Following the shRNA transfection of the GCs with *NDUFAB1* or *GABRA1* silence, a variation in cell proliferation was determined by EdU (5′-Ethynyl-2′- deoxyuridine) incorporation assay using the Cell-Light EdU imaging kit (RiboBio, Guangzhou, China) according to the manufacturer’s protocol. Each experiment was performed in triplicate and repeated five times. The number of EdU-positive cells was expressed as a percentage and calculated relative to the total number of cells (positive cells/total cells in one field) as previously described [8]. The ratio of positive cells was calculated from the average of the five group values.

### Cell apoptosis assay

The apoptosis of GCs was determined by flow cytometry (BD Biosciences, USA) using the Annexin V- Allophycocyanin(APC)/7-amino-actinomycin D (AAD) Apoptosis Detection Kit (KeyGEN, Nanjing, China), in accordance with the manufacturer’s protocols. The apoptosis rate of GCs was estimated, i.e., total number of apoptotic cells comprises the number of cells in the right upper quadrant (Q1-UR) and number of cells in the right lower quadrant (Q1-LR), and analyzed using FlowJo v7.6 software (Stanford University, Stanford, CA, USA). Each experiment was carried out for at least three times.

### Statistical analysis

Statistical analysis was fulfilled using the SPSS12.0 software package [95]. Data were analyzed by executing a Student’s *t-*test for comparisons between the RNA-Sequencing and RT-qPCR determination after confirmation of normal distributions for non-parametric analysis. Values were presented as mean ± SEM and bars with superscript symbols that indicate the significant difference compared with control groups at *** *p* < 0.001, ** *p* < 0.01, * *p* < 0.05.

## Supplementary Information

**Additional file 1: Supplementary Table S1.** Results of the cleaned data of reads filtered and the reads mapped. **Supplementary Table S2.** Construction frame of lentiviral vectors against *NDUFAB1* and *GABRA1* genes. **Supplementary Table S3.** Antibodies used for western blot analysis. **Supplementary Figure S1.** The oxidative phosphorylation pathway (gga: 00190) and its components. **Supplementary Figure S2.** The neuroactive ligand-receptor interaction pathway (gga: 04080) and its components. **Supplementary Figure S3.** The images show the original gels of western blot in the Fig. 6B and Fig. 6E. **Supplementary Figure S4.** The images show the original gels of western blot in the Fig. 8B and Fig. 8E.

## Data Availability

The relevant datasets have been published by our group and are available at PRJNA669967 (https://dataview.ncbi.nlm.nih.gov/object/PRJNA669967?reviewer=r1qgd76c8kfv73u2c0r3ruc4vm) and PRJNA670553 (https://dataview.ncbi.nlm.nih.gov/object/PRJNA670553?reviewer=3fu2up8dl58afnlahsue09njcp) in NCBI [23].

## Abbreviations

NDUFAB1: NADH: ubiquinone oxidoreductase subunit AB
GABRA1: Gamma-aminobutyric acid type A receptor alpha1 subunit
STAR: Steroidogenic-related enzymes steroidogenic acute regulatory protein
HSD17B1: Hydroxysteroid (17beta) dehydrogenase 1
P450scc/CYP11A1: Cytochrome P450 side-chain cleavage
P4: Progesterone
E2: Estradiol
AMH: Anti-müllerian hormone
CCND1: Cyclin D1
CASP3: Caspase-3
Bcl-2: B-cell lymphoma-2
GO: Gene ontology
KEGG: Kyoto encyclopedia of genes and genomes
GWF: Growing white follicle
SYF: Small yellow follicle
LYF: Large yellow follicle
DEG: Differentially expressed gene
GC: Granulosa cell
JB: Jilin Black chicken
LB: Lohmann Brown chicken
siRNA: Small interfering RNA
shRNA: Small hairpin RNA
EdU: 5′-Ethynyl-2′- deoxyuridine
APC: Annexin V- Allophycocyanin
AAD: 7-amino-actinomycin D
AF: Age at first egg
BW: Body weight
EW: Egg weight
HH: Hen-housed egg production
EN: Egg numbers per hen
cAMP: Adenosine-3′,5′-cyclic monophosphate
CAM: Cell adhesion molecule
AMPK: AMP-activated protein kinase
FSH: Follicle stimulating hormone (FSH)
FSHR: FSH receptor
PACAP: Adenylate cyclase-activating polypeptide
VIP: Vasoactive intestinal peptide
PAC1: PACAP type I receptor
VIPR: VIP receptor
TC: Theca cell
ADRB2: β2 adrenergic receptor
PKA: Protein kinase A
PKC: Protein kinase C
PLC: Phospholipase C
CREB: cAMP response element-binding protein
PCOS: Polycystic ovary syndrome
ACP: Acyl carrier protein
