# Supplementary material for: Transcriptome comparative analysis of ovarian follicles reveals the key genes and signaling pathways implicated in hen egg production
Source: BMC Genomics. 2021 Dec 15;22:899. doi: 10.1186/s12864-021-08213-w (PMC8672471; doi:10.1186/s12864-021-08213-w)

Transcriptome comparative analysis of ovarian follicles reveals the key genes and signaling pathways implicated in hen egg production

Xue Sun^1,2†^, Xiaoxia Chen^1,2†^, Jinghua Zhao^1,2^, Chang Ma^1,2^, Chunchi Yan^1,2^, Simushi Liswaniso^1,2^, Rifu Xu^1,2*^ and Ning Qin^1,2*^

^1^Joint Laboratory of Modern Agricultural Technology International Cooperation, Ministry of Education, Jilin Agricultural University, Changchun 130118, China

^2^Department of Animal Genetics, Breeding and Reproduction, College of Animal Science and Technology, Jilin Agricultural University, Changchun 130118, China

*Correspondence: [poultryxu@jlau.edu.cn](mailto:poultryxu@jlau.edu.cn); ningqin@jlau.edu.cn

^†^Xue Sun and Xiaoxia Chen contributed equally to this work.

**Supplementary Information：**

**Supplementary Table S1** Results of the cleaned data of reads filtered and the reads mapped. (DOCX)

**Supplementary Table S2** Construction frame of lentiviral vectors against NDUFAB1 and GABRA1 genes. (DOCX)

**Supplementary Table S3** Antibodies used for western blot analysis. (DOCX)

**Supplementary Figure S1** The oxidative phosphorylation pathway (gga: 00190) and its components. (TIFF)

**Supplementary Figure S2** The neuroactive ligand-receptor interaction pathway (gga: 04080) and its components. (TIFF)

**Supplementary Figure S3** The images show the original gels of western blot in the Figure 6B and Figure 6E. (TIFF)

**Supplementary Figure S4** The images show the original gels of western blot in the Figure 8B and Figure 8E. (TIFF)

**Table S1** Results of the cleaned data of reads filtered and the reads mapped

| Sample | Clean reads No. | Clean data (bp) | Clean reads (%) | Clean data (%) | Total mapped | Uniquely mapped |
| --- | --- | --- | --- | --- | --- | --- |
| L11 | 43457970 | 6542567926 | 99.36 | 99.07 | 36989154 (85.11%) | 35608927 (96.27%) |
| L12 | 41750694 | 6285289046 | 99.41 | 99.11 | 35669568 (85.43%) | 34285668 (96.12%) |
| L13 | 46722392 | 7033912150 | 99.40 | 99.10 | 40031782 (85.68%) | 38296573 (95.67%) |
| L21 | 45674436 | 6875717490 | 99.40 | 99.09 | 39059946 (85.52%) | 37456729 (95.90%) |
| L22 | 42199084 | 6352676798 | 99.45 | 99.15 | 36169975 (85.71%) | 34632945 (95.75%) |
| L23 | 42011124 | 6323848904 | 99.45 | 99.14 | 35837904 (85.31%) | 34024629 (94.94%) |
| L31 | 51902968 | 7812567910 | 99.54 | 99.23 | 44549945 (85.83%) | 42859977 (96.21%) |
| L32 | 44559406 | 6708743716 | 99.56 | 99.27 | 38209439 (85.75%) | 36601131 (95.79%) |
| L33 | 41417292 | 6236982404 | 99.52 | 99.25 | 35483457 (85.67%) | 33622013 (94.75%) |
| J11 | 52955756 | 7973000446 | 99.50 | 99.21 | 45212064 (85.38%) | 43403482 (96.00%) |
| J12 | 48010434 | 7230278376 | 99.32 | 99.06 | 41237426 (85.89%) | 39484763 (95.75%) |
| J13 | 46024668 | 6932185704 | 99.32 | 99.07 | 39390948 (85.59%) | 37573391 (95.39%) |
| J21 | 44326682 | 6676460600 | 99.32 | 99.07 | 38338558 (86.49%) | 36895375 (96.24%) |
| J22 | 47741962 | 7190302714 | 99.34 | 99.08 | 40955171 (85.78%) | 39506326 (96.46%) |
| J23 | 48609428 | 7321313074 | 99.21 | 98.95 | 41443727 (85.26%) | 39660637 (95.70%) |
| J31 | 41657196 | 6274749870 | 99.19 | 98.94 | 35554659 (85.35%) | 34123759 (95.98%) |
| J32 | 43542932 | 6557837122 | 99.17 | 98.91 | 37123950 (85.26%) | 35490354 (95.60%) |
| J33 | 45189634 | 6806288406 | 99.21 | 98.96 | 38683810 (85.60%) | 36852463 (95.27%) |

Notes: L and J, indicate samples harvested from LB and JB laying hens. First digits, 1, 2 and 3 mark the follicle type of GWF, SYF and LYF. Second digits, 1, 2 and 3 indicate follicles are from different hens.

| Lentiviral  vector | 5′flanking Stem Loop Stem 3′flanking |
| --- | --- |
| sh-*NDUFAB1* | .GGATCCCCACAAGAGATAGTAGATT**TTCAAGAGA**AATCTACTATCTCTTGTGGTTTTTT*CTCGAG*GAATTC |
|  | GAATTC*CTCGAG*AAAAAACCACAAGAGATAGTAGATT**TTCAAGAGA**AATCTACTATCTCTTGTGGGGATCC |
| sh-*GABRA1* | .GGATCCGCAGAATGTCCAATGCATT**TTCAAGAGA**AATGCATTGGACATTCTGCTTTTTT*CTCGAG*GAATTC |
|  | GAATTC*CTCGAG*AAAAAAGCAGAATGTCCAATGCATT**TTCAAGAGA**AATGCATTGGACATTCTGCGGATCC |
| shRNA-NC | .GGATCCTTCTCCGAACGTGTCACGT**TTCAAGAGA**ACGTGACACGTTCGGAGAATTTTTT*CTCGAG*GAATTC |
|  | GAATTC*CTCGAG*AAAAAATTCTCCGAACGTGTCACGT**TTCAAGAGA**ACGTGACACGTTCGGAGAAGGATCC |

**Table S2** Construction frame of lentiviral vectors against *NDUFAB1* and *GABRA1* genes

Note: Underlined nucleotides indicate the *BamHI* restriction endounclease site, the dot line indicates the sequence of the *EcoRI* restriction site, and the italics nucleotides mark the *XhoI* restriction site. The bold nucleotides present the loop region at RNA level.

**Table S3** Antibodies used for western blot analysis

| **Protein target** | **Primary**  **antibody** | **Dilution used** | **Antibody type** | **Secondary**  **antibody** | **Dilution used** | **Originated** |
| --- | --- | --- | --- | --- | --- | --- |
| cNDUFAB1 | Rabbit anti- cNDUFAB1 | 1/20000 | Monoclonal | anti-rabbit IgG | 1/50000 | Abcam Cambridge, MA, USA |
| cGABRA1 | Rabbit anti-  cGABRA1 | 1/2000 | polyclonal | anti-rabbit IgG | 1/50000 | Proteintech,WuHan China |
| cSTAR | Rabbit anti- cSTAR | 1/1000 | polyclonal | anti-rabbit IgG | 1/50000 | Abcam Cambridge, MA, USA |
| cCYP11A1 | Rabbit anti- cCYP11A1 | 1/1000 | polyclonal | anti-rabbit IgG | 1/50000 | Abcam Cambridge, MA, USA |
| cCCND1 | Rabbit anti-  cCCND1 | 1/5000 | Monoclonal | anti-rabbit IgG | 1/50000 | Abcam Cambridge, MA, USA |
| cBCL-2 | Mouse anti- cBCL-2 | 1/500 | Monoclonal | anti-mouse IgG | 1/50000 | BD Biosciences, Palo Alto, CA |
| cCaspase-3 | Rabbit anti-  cCaspase-3 | 1/1000 | polyclonal | anti-rabbit IgG | 1/50000 | Enzo Farmingdale, NY, USA |
| cβ-actin | Mouse anti- cβ-actin | 1/500 | Monoclonal | anti-mouse IgG | 1/50000 | Boster Biological Technology, China |

Note: Source of the antibodies against the chicken protein/peptide target list in this table. Antibody concentration: 1μg/ul, size: 100 ul.

**Figure S1** The oxidative phosphorylation pathway (gga: 00190) and its components. (TIFF)


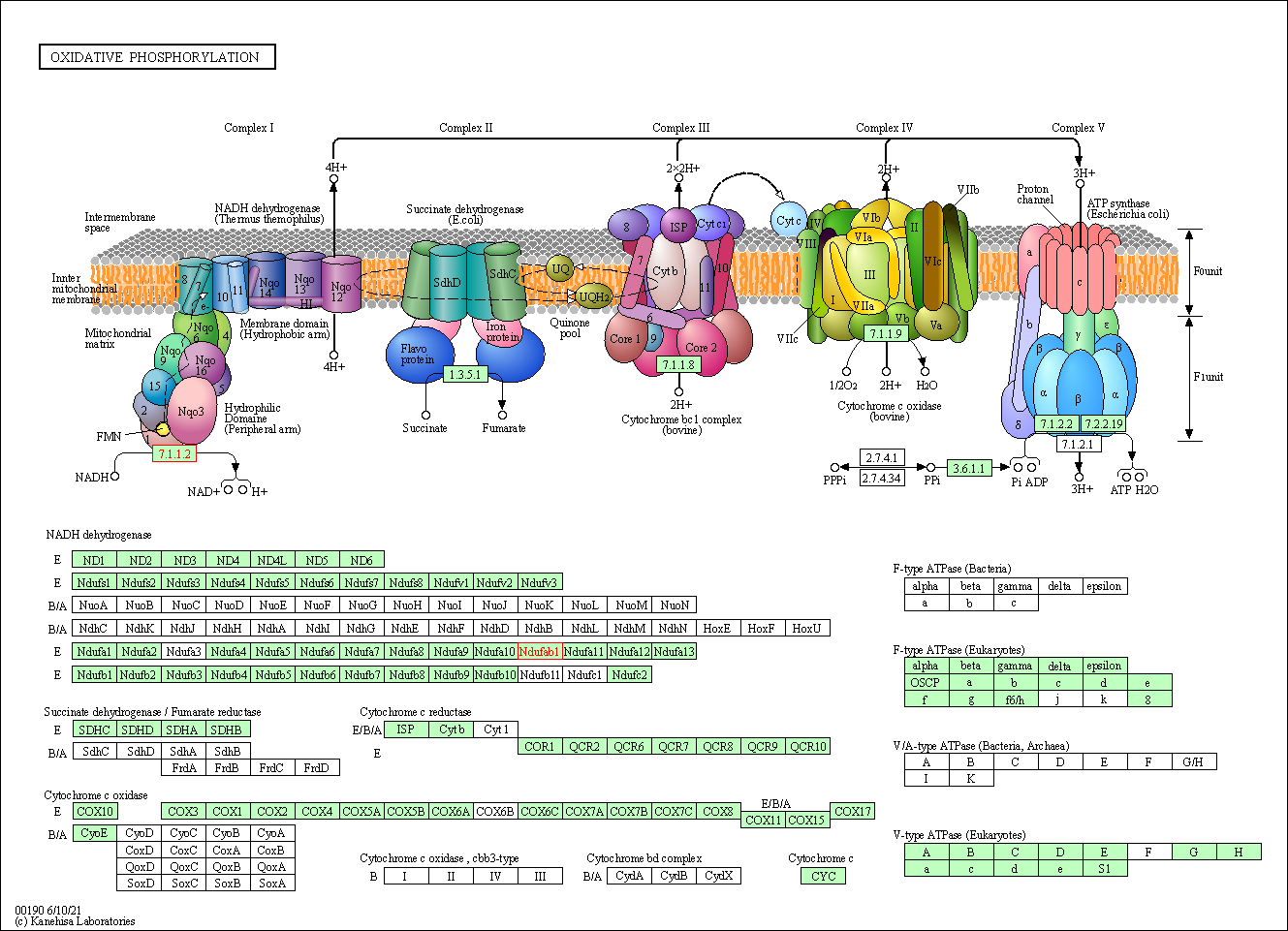


**Figure S2** The neuroactive ligand-receptor interaction pathway (gga: 04080) and its components. (TIFF)


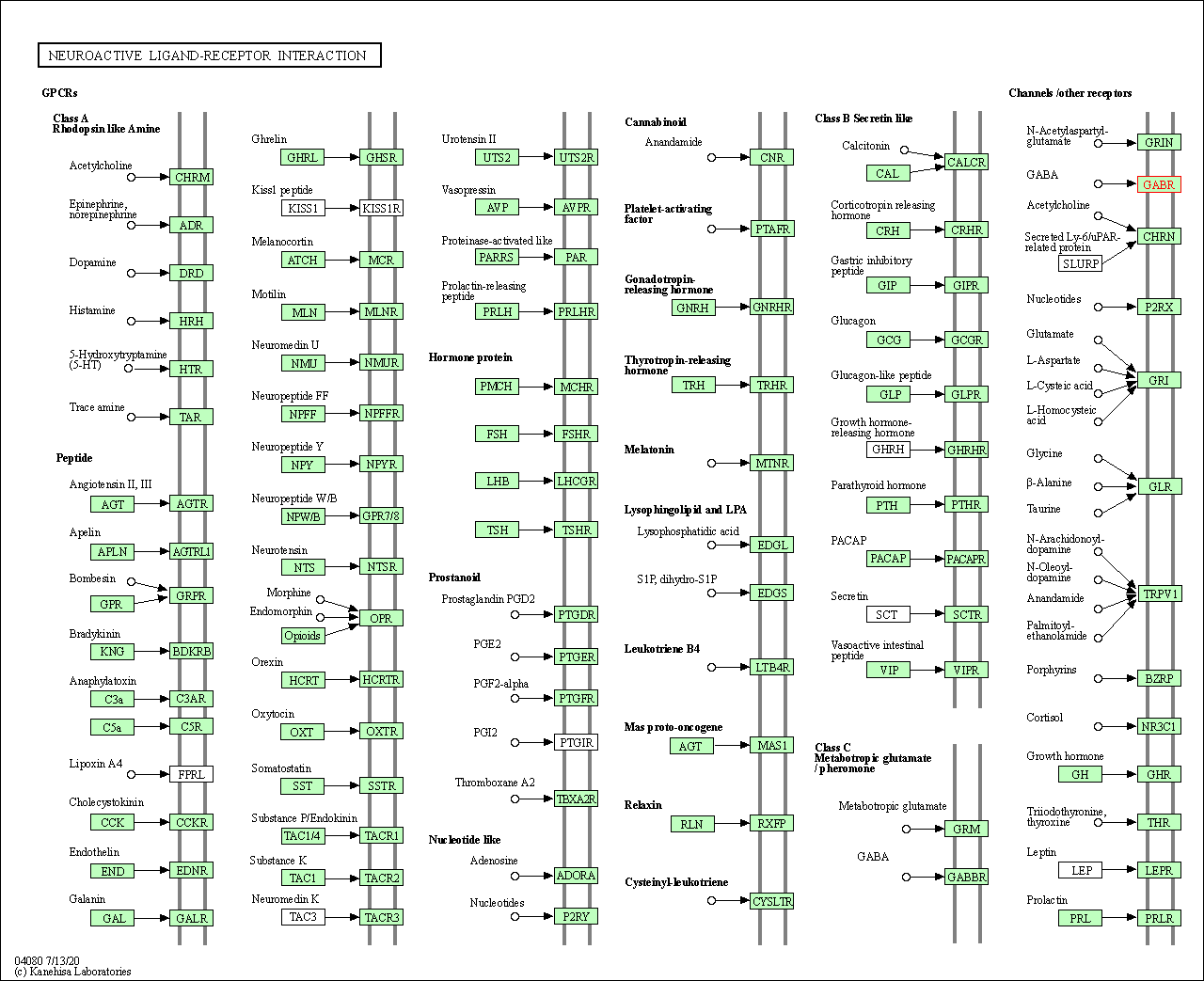


**Figure S3** The images show the original gels of western blot in the Figure 6B and Figure 6E. (TIFF)


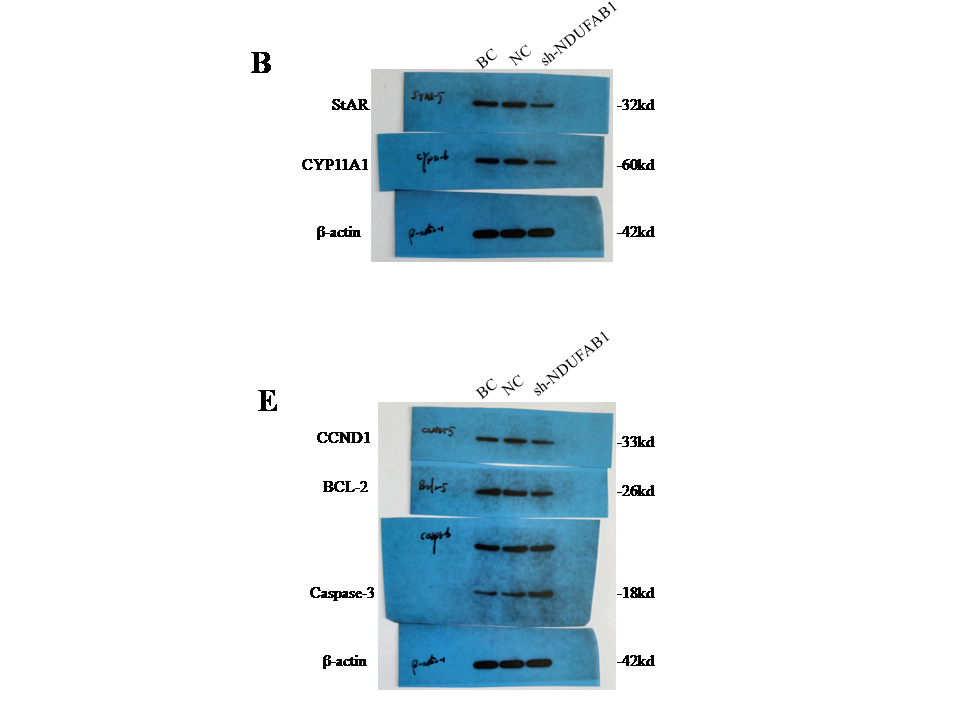


**Figure S4** The images show the original gels of western blot in the Figure 8B and Figure 8E. (TIFF)


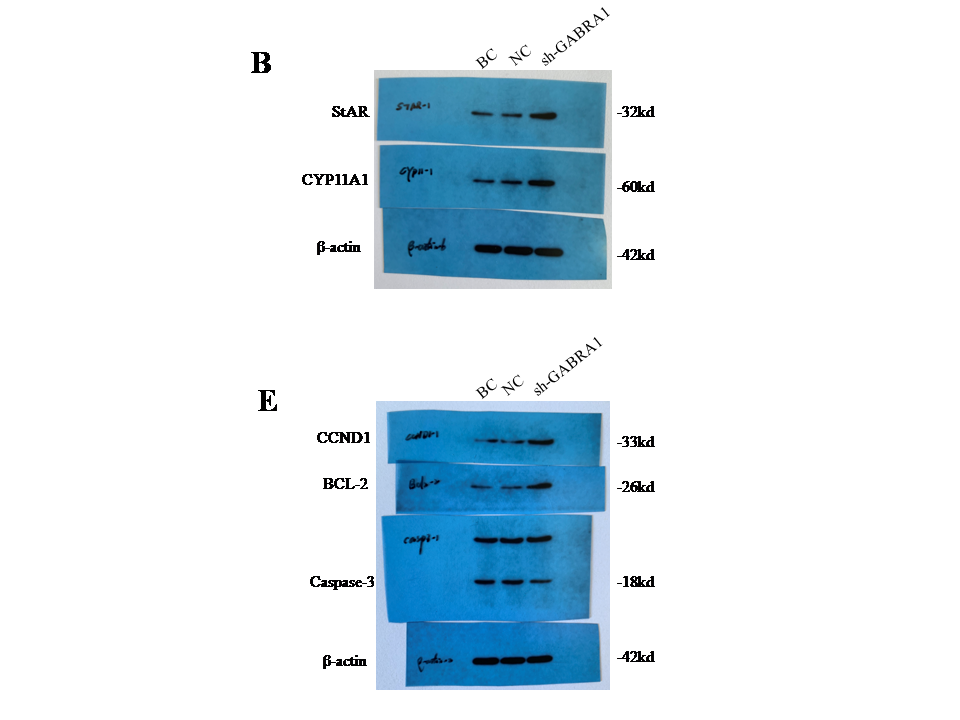

Supplement: Supplementary file 1 — Additional file 1: Supplementary Table S1. Results of the cleaned data of reads filtered and the reads mapped. Supplementary Table S2. Construction frame of lentiviral vectors against NDUFAB1 and GABRA1 genes. Supplementary Table S3. Antibodies used for western blot analysis. Supplementary Figure S1. The oxidative phosphorylation pathway (gga: 00190) and its components. Supplementary Figure S2. The neuroactive ligand-receptor interaction pathway (gga: 04080) and its components. Supplementary Figure S3. The images show the original gels of western blot in the Fig. 6B and Fig. 6E. Supplementary Figure S4. The images show the original gels of western blot in the Fig. 8B and Fig. 8E. [file 12864_2021_8213_MOESM1_ESM.docx]
